# Mpi-driven N-glycosylation orchestrates mucin O-glycosylation and intestinal homeostasis

**DOI:** 10.1038/s41467-026-73100-5

**Published:** 2026-05-18

**Authors:** Avishek Roy, Steve Meregini, Hye-Jeong Cho, Zhenglan Chen, Aariz Zaki, Tandav Argula, Bruce Beutler, Jeffrey A. SoRelle

**Affiliations:** 1 Department of Pathology, Division of Genomic and Molecular Pathology, University of Texas Southwestern Medical Center, Dallas, TX USA; 2 Center for Genetics of Host Defense, University of Texas Southwestern Medical Center, Dallas, TX USA; 3 Department of Pediatrics, Division of Allergy and Immunology, University of Texas Southwestern Medical Center, Dallas, TX USA

**Keywords:** Mucosal immunology, Inflammatory bowel disease, Disease genetics

## Abstract

The intestinal mucus barrier physically separates the epithelium from the dense microbial community of the gut and is essential for intestinal homeostasis. The principal component, the gel-forming mucin MUC2, is extensively glycosylated, yet how different classes of glycans regulate mucin function remains unclear. Here we show that N-glycosylation is required for proper MUC2 maturation and mucus barrier integrity. Using mouse models with hypomorphic or intestinal epithelial–specific loss of the mannose-generating enzyme MPI, which is required for N-glycan synthesis, we find that impaired N-glycosylation disrupts mucin processing and secretion. MPI deficiency results in severe susceptibility to dextran sulfate sodium–induced colitis or spontaneous intestinal inflammation, accompanied by endoplasmic reticulum stress, microbial dysbiosis, and defects in Paneth cells. These findings demonstrate N-glycosylation is critical for mucus barrier function and reveal an unexpected link between N-glycosylation and intestinal inflammatory disease.

## Abstract (web summary)

Authors show that N-glycosylation is required for proper MUC2 maturation and mucus barrier integrity. They find impaired N-glycosylation disrupts mucin processing and secretion in mouse models deficient in the mannose-generating enzyme MPI, resulting in severe intestinal pathology.

## Introduction

Glycosylation is a major post-translational modification that attaches sugar molecules to protein and other macromolecules^1^. Over half of all proteins involved in essential cellular processes are glycosylated, underscoring the importance to health and disease^2^. Mucins are one of the important classes of heavily glycosylated proteins. These heavily O-glycosylated proteins form a protective barrier along mucosal surfaces, including the gut. Here, they not only shield epithelial cells but also shape the microbiome by feeding commensal bacteria in the mucus layer^3^. N-and O- linked glycans are added to mucin proteins in the endoplasmic reticulum (ER) and Golgi apparatus, respectively^4,5^. Mucin 2 (MUC2), the predominant secreted mucin in the gut, requires proper glycosylation for its gel-forming properties and for maintaining an intact mucus layer^6,7^.

Defects in glycosylation occur in rare inherited conditions known as Congenital Disorders of Glycosylation (CDG). These disorders^8^ have provided mechanistic insights into systemic roles of glycans^9^. One such disorder, MPI-CDG (CDG-1b), results from mutations in mannose phosphate isomerase (*MPI*), an enzyme that catalyzes the interconversion of fructose-6-phosphate to mannose-6-phosphate, a rate-limiting step in N-linked glycosylation^7,8^. In humans, MPI-CDG is very rare (< 1:1,000,000)^9^ and characterized by protein-losing enteropathy (PLE), chronic diarrhea, cirrhosis, cyclic vomiting, anemia, and hyperinsulinemic hypoglycemia^10,11^. Remarkably, these symptoms can be corrected using mannose supplementation^12,13^, to bypass the *MPI*-dependent step in mannose synthesis.

Our previous work identified a hypomorphic *Mpi* allele (*benadrl*) through a forward genetic screen in mice. The *N*-ethyl-*N*-nitrosourea (ENU)-induced point mutation^14–16^, later reproduced by CRISPR knock-in, reduced Mpi enzymatic activity to 5% of normal due to weaker substrate binding affinity^16^. Mice carrying this allele exhibit phenotypes attributable to intestinal hypo-N-glycosylation, such as diarrhea and loss of goblet cells^9,16,17^. These findings suggest Mpi loss disrupts mucin maturation and gut barrier function.

To directly test this hypothesis, we generated the first intestinal epithelial- specific *Mpi* knockout mice (Mpi^ΔVillin^). These Mpi^ΔVillin^ mice exhibit spontaneous colitis with symptoms that resemble ulcerative colitis in humans. Our analyses indicate *Mpi* deficiency impairs Muc2 synthesis and secretion, leading to compromised mucus barrier integrity and dysbiosis. Furthermore, this strain exhibits increased ER stress, fewer mitochondria, loss of Paneth cell granulation, and decreased fatty acid enzymes. In this study, we aim to define how Mpi-mediated N-glycosylation regulates mucin biology and gut barrier function, and how disruptions in these pathways contribute to intestinal inflammation.

## Results

### Mpi mice are susceptible to DSS-induced colitis

**Fig. 1:**
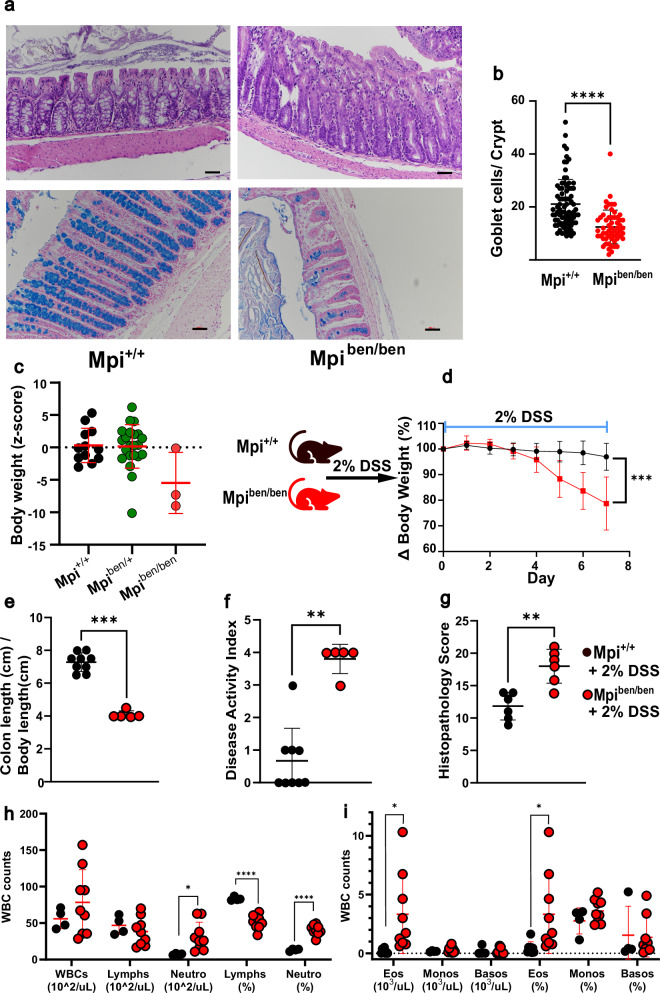
*Mpi* deficiency reduces colon goblet cells with susceptibility to DSS-induced colitis. **a** Representative colon sections stained with Haematoxylin and Eosin (top panel) and Alcian Blue (bottom panel) for colons Mpi^+/+^ and Mpi^ben/ben^ mice. Scale bars = 100 μm. **b** Quantification of goblet cells/crypt for Mpi^+/+^ and benadrl mice (villi *n* = 85 for Mpi^+/+^ and *n* = 57 for Mpi^ben/ben^, collected from *n* = 9 Mpi^+/+^ and *n* = 7 Mpi^ben/ben^ mice). **c** ENU-mutagenized Mpi^ben/ben^ mice were quantitatively smaller in size compared to littermates. Z-score body weight analysis adjusted for age and sex (*n* = 12, 22, 3). **d** Weight loss analysis of Mpi^+/+^ and Mpi^ben/ben^ mice after treatment with 2% DSS (*n* = 7 mice each group, representative of two independent experiments). **e–g** Colon length, disease activity index and histopathological score 7 days after DSS treatment (*n* = 9 for Mpi^+/+^ and *n* = 5 for Mpi^ben/ben^). For this figure (**g**) *P*-value = 0.0065. **h**, **i** Absolute and relative numbers of white blood cells (WBCs), lymphocytes (Lymphs), neutrophils (Neutro), eosinophils (Eos), monocytes (Monos), and basophils (Basos) in peripheral blood. For this figure (**h**) (*n* = 4 for WT and *n* = 9 for Mpi^ben/ben^, independent biological replicates; *P* value = 0.00056 and 0.0001 for Lymphs (%) and Neutro (%))and for this figure (**i**) (*n* = 8 for WT and *n* = 9 for Mpi^ben/ben^, independent biological replicates) (*P* value = 0.0169 for Eos (10^3^/uL) and *P* value = 0.030 for Eos (%); **P < 0.01, ***P < 0.001, and ****P < 0.0001). **c** analyzed using Mann–Whitney test, (**d**, **e–g**) analyzed using student *t*-test. WT wild type, DSS Dextran Sodium Sulfate. Data expressed as means ± s.d. and significance was determined as unpaired Student *t*-test for this figure (**b**, **d**–**i**). **d** created in BioRender. Sorelle, J. (2026) https://BioRender.com/alne1e2.

During a forward genetic screen using ENU mutagenesis, we identified a mouse strain exhibiting an allergic phenotype and small body size with recessive inheritance (Supplementary Fig. 1a and Fig. 1a*p* = 1.6 × 10^−12^), which we named *benadrl*. This strain mapped to a missense variant p.H54R in the *Mpi* gene. We have previously described this strain^16^, which is notable for recreating most clinical features of the human congenital disorder of glycosylation MPI-CDG, including congenital diarrhea. To better understand the cellular mechanisms of Mpi loss in the intestine, we compared *MPI* expression in open-source single-cell RNA-seq data sets of human tissues. *MPI* RNA was highest in proximal enterocytes, but goblet cells, Paneth cells, and distal enterocytes each expressed comparable levels of MPI RNA (graphic user interface of proteinatlas.org used)^18^. Colon histopathology demonstrated a significant reduction in goblet cells confirmed by Alcian blue staining of glycoproteins (Fig. 1b, c, *p* < 0.0001). This phenotype is the most severe of any reported goblet cell genetic defect, including those seen in other glycosylation-related genes (Tvp23b, Yipf6, C1galt1, C3Gnt)^19–22^ found by our group. The phenotype resembles the Muc2 KO strain,^16,23^, indicating *Mpi’s* essential role in Muc2 production and goblet cell homeostasis.

Based on these findings, we hypothesized that *benadrl* mice would be susceptible to DSS-induced colitis. In fact, *benadrl* mice had a strong response to 2% DSS, requiring euthanasia by day 7 (Fig. 1d, *p* < 0.001). Disease severity was assessed by multiple metrics, which demonstrated worse colitis in *benadrl* mice. The colon length (normalized to body length) was significantly shorter (*p* < 0.0005), along with a significantly elevated disease activity index (*p* < 0.0005) and higher histopathology score (Supplementary Fig. 1b and Fig. 1e–g) in *benadrl* mice. Also, *Mpi* deficiency led to significantly higher levels of peripheral blood neutrophils and eosinophils in Mpi^ben/ben^ at baseline (*p* < 0.05, Fig. 1h, i).

### Intestinal loss of Mpi causes spontaneous colitis

**Fig. 2:**
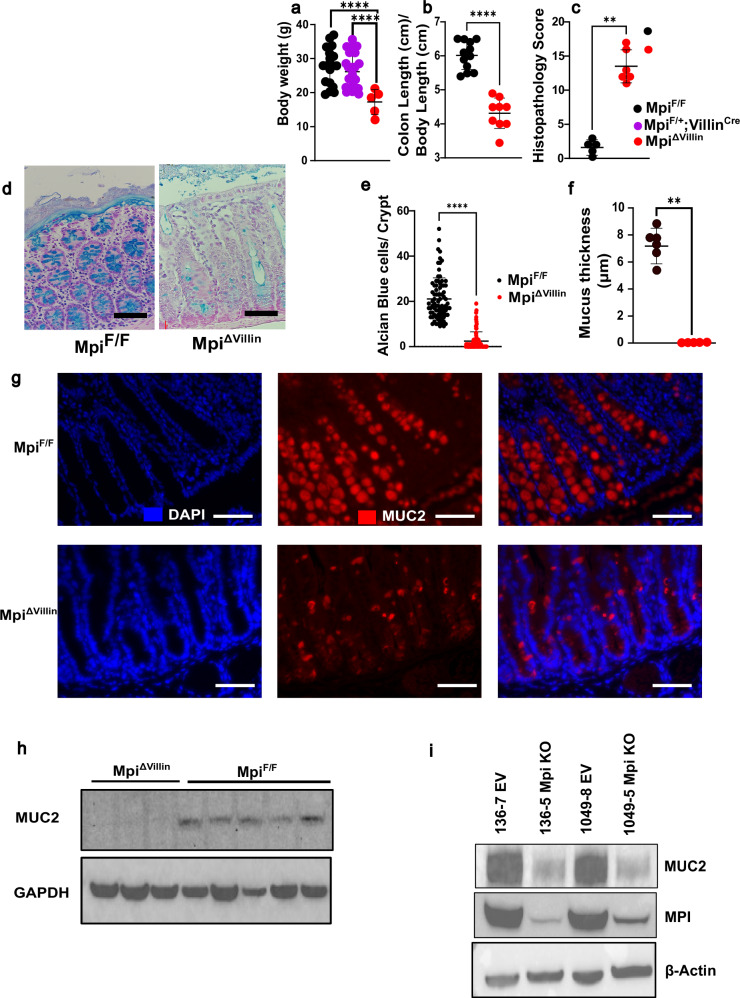
Intestinal-specific Mpi knockout causes severe colon pathology. **a** Weight of Mpi^F/F^, Mpi^F/+^ and Mpi^ΔVillin^ mice 4 weeks after birth (*n* = 14 for Mpi^F/F^, *n* = 14 for Mpi^F/+^ and *n* = 5 for Mpi^ΔVillin^; *P* value = 0.0005 for Mpi^F/F^ vs Mpi^ΔVillin^ and *P* value = 0.0002 for Mpi^F/+^ vs Mpi^ΔVillin^). **b** Colonic lengths of mice 4 weeks after birth (*n* = 12 for Mpi^F/F^ and *n* = 9 for Mpi^ΔVillin^). **c** Histopathologic score of colons in Mpi^F/F^ and Mpi^ΔVillin^ mice (*n* = 5, 6, *P* value = 0.001respectively). **d** Representative Alcian Blue stained sections of colons Mpi^F/F^ vs Mpi^ΔVillin^. **e** Quantitative analysis of goblet cells/crypt across colon for Mpi^F/F^ vs Mpi^ΔVillin^ (*n* = 5 independent mice each group and *n* = 85 crypts each group). **f** Quantitative analysis of Mucus thickness (µm) Mpi^F/F^ vs Mpi^ΔVillin^ (*n* = 5 mice for each group, *P* value = 0.0079). **g** Representative Muc2 immunohistochemistry from Mpi^F/F^ vs Mpi^ΔVillin^ colons (repeated 5 times). **h** Immunoblot of mucin-2 in colon lysates from Mpi^ΔVillin^ (*n* = 3) and Mpi^F/F^ animals (*n* = 5) (representative image of three experiments). **i** Immunoblot of MUC2, MPI, and β- Actin (*MUC2 Mucin 2, MPI Mannose Phosphate Isomerase*, and *β-Actin beta Actin*) in LS174T WT and Mpi KO cells -136-7 EV, 136-5 Mpi KO, 1049-8 EV, and 1049-5 Mpi KO (where EV Empty Vector), representative image of 3 independent experiments. Mpi KO cell lines are designated: 136-8 for the 8th clone of a CRISPR targeting *Mpi* exon 2 (cDNA position 136) and 1049-5 for the 5th clone of a CRISPR targeting *Mpi* exon 7 (cDNA position 1049). **e** Mann-Whitney test (**a**–**c**, and **h**) Student *t*-test (**P < *0.01*, ***P < *0.001*, and ****P < *0.0001*). **a–c**, **e**, **f** Data expressed as means ± s.d. and significance was determined as unpaired Student *t*-test. **c**–**e** statistical significance was calculated using two-tailed Mann–Whitney test. Data is representative or aggregated from at least 3 independent experiments. Scale bars (**f**, **i**) are 50 μm in length.

To test whether the colitis phenotype was intrinsic to intestinal epithelial cells, we created the Mpi^ΔVillin^ mouse strain. This strain was previously described^16^ to excise exon 3 of the *Mpi* gene by flanking loxP sequences in cells expressing the Cre recombinase under the control of the Vil1 promoter. Mpi^ΔVillin^ mice were born at 50% of the expected Mendelian ratio of 25% (Mpi^f/f^ × Mpi^f/+^; Villin^Cre^) and were not fertile. In the barrier facility, Mpi^ΔVillin^ mice survived well through 4 months, but in conventional housing, most died in 6–8 weeks. The Mpi^ΔVillin^ mice developed spontaneous colitis with a much smaller body size and shortened colon (Fig. 2a, b and Supplementary Fig. 2). The mice also show higher histopathological score (Fig. 2c) with symptoms of rectal bleeding. The mucus layer, visualized by Alcian blue staining, is nearly absent decreasing from 8 μm in WT to undetectable levels (0 μm) in Mpi^ΔVillin^ mice (Fig. 2d–f). Both immunohistochemistry (Fig. 2g) and immunoblotting of whole large intestine lysates (Fig. 2h) demonstrated reduced Mucin 2 (Muc2) protein, confirming mucus loss. To confirm that MPI loss impairs mucin production, at the cellular level, we generated *MPI* knockout LS174T colonic epithelial mucus-secreting cell lines, which exhibited markedly reduced MUC2 expression across independent clones (Fig. 2i).

### Intestinal loss of Mpi results in spontaneous colitis

**Fig. 3:**
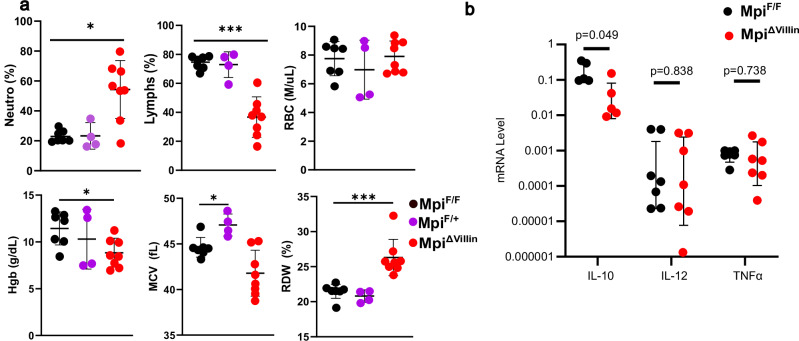
Loss of intestinal glycosylation causes Inflammation. **a** Whole blood count in Mpi^F/F^, Mpi^F/+^; and Mpi^ΔVillin^ (*n* = 7 for Mpi^F/F^, *n* = 4 for Mpi^F/+^ and *n* = 7 for Mpi^ΔVillin^, *P* value = 0.0006for Neutro %, *P* value = 0.0103 for Hgb, *P* value = 0.0182 for MCV and *P* value = 0.0001 for RDW%). **b** Gapdh normalized mRNA expression of IL-10, IL-12, and TNFα in colon tissue between Mpi^F/F^ and Mpi^ΔVillin^ 4-week-old mice (*n* = 5 for both Mpi^F/F^ and Mpi^ΔVillin^ for IL-10 mRNA expression and *n* = 7 for both the groups for IL-12 and TNFα mRNA expression). Data expressed as means ± s.d. and significance was determined using One-Way ANOVA with Tukey post-hoc test for multiple comparisons (**a**) or Mann–Whitney test (**b**). Data is aggregated from 3 independent experiments. (*P < 0.05, **P < 0.01, ***P < 0.001). Neutro Neutrophil, Lymphs lymphocytes, RBC red blood cell count, Hgb hemoglobin, MCV mean corpuscular volume, RDW red cell distribution width, IL-10 interleukin 10, IL-12 interleukin 12, TNFα tumor necrosis factor alpha.

The inflammatory nature of the colitis phenotype in Mpi^ΔVillin^ mice was examined by cytokine and cellular analysis. Peripheral blood counts indicate systemic acute inflammation, evidenced by elevated neutrophils. In addition, Mpi^ΔVillin^ mice developed microcytic anemia with increased red cell distribution width (RDW) consistent with a reactive anemia, possibly linked to malabsorption or acute inflammation (Fig. 3a). Pro-inflammatory cytokine IL-12 and TNF-α mRNA expression were not increased in Mpi^ΔVillin^ colons (*p* > 0.05, Fig. 3b), whereas the anti-inflammatory cytokine IL-10 was significantly reduced compared to the Mpi^F/F^ colon (*p* = 0.04, Mann–Whitney test, Fig. 3b).

### ER stress is induced by Mpi deficiency

**Fig. 4:**
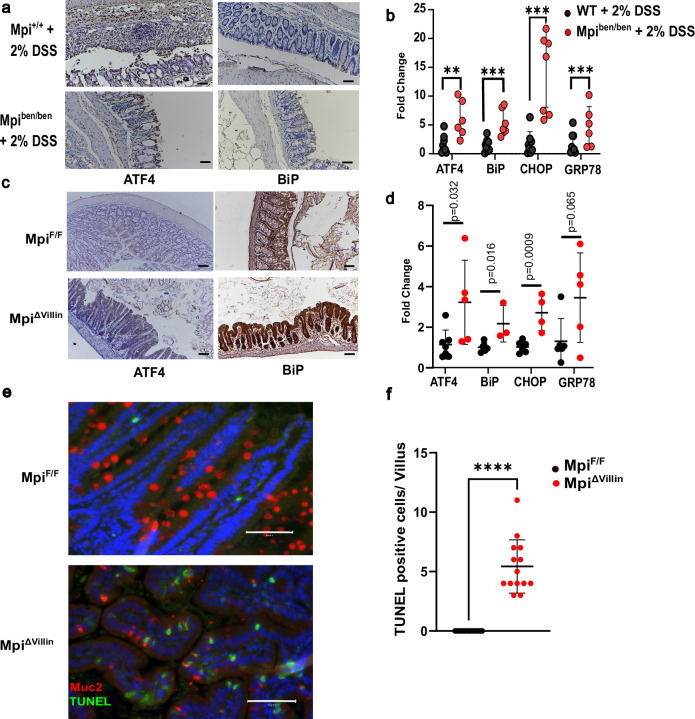
Abnormal ER stress response. **a** Representative image from Immunohistochemistry for ER stress proteins ATF4 and BiP across colons of Mpi^+/+^ vs Mpi^ben/ben^ 7 days after 2% DSS treatment (repeated three times). Scale bars are 100 μm in length. **b** Relative mRNA expression (ΔΔCT) of ER stress response specific genes- *ATF4, BiP, CHOP*, and *Grp78* (*n* = 6 independent mice, *P* value = 0.0079 for ATF4, *P* value = 0.0013 for BiP, *P* value = 0.0013 for CHOP, and *P* value = 0.0426 for GRP78. *P < 0.05, **P < 0.01, ***P < 0.001, ****P < 0.0001). **c** Representative image from Immunohistochemistry for ER stress proteins ATF4 and BiP across colons of Mpi^F/F^ vs Mpi^ΔVillin^ (repeated three times). **d** Relative mRNA expression (ΔΔCT) of ER stress response specific genes (normalized to GAPDH, *n* = 7 and *n* = 5 for ATF4, *n* = 6 and *n* = 3 for BiP, *n* = 7 and *n* = 4 for CHOP, *n* = 6 and *n* = 5 for GRP94 mice, each for Mpi^F/F^ and Mpi^ΔVillin^, *P < 0.05). **e** Immunofluorescence of TUNEL (green) and Muc2 (red) from Mpi^F/F^ and Mpi^ΔVillin^ ileum samples (*n* = 3 mice each). Scale bars are 50 μm in length. **f** Quantitative analysis of TUNEL positive cells/crypt Mpi^F/F^ and Mpi^ΔVillin^ (*n* = 3 mice per group). **b**, **d**, and **f** Data are expressed as means ± s.d. and significance was determined using Mann–Whitney unpaired Student *t*-test. Data is representative of at least 3 independent experiments. **b**, **d**, and **f** Student *t*-test. **b**, **d**, and **f** – statistical significance was calculated using two-tailed Unpaired Mann–Whitney Student *t*-test. ATF4 Activating Transcription Factor 4, BiP Immunoglobulin binding protein, CHOP C/EBP homologous protein, Grp78 Glucose regulated protein 78, Grp94 Glucose regulated protein 94.

We considered the profound loss of protective mucus could be related to protein misfolding and subsequent ER stress, which is known to impact intestinal homeostasis^2^. While O-glycosylation makes up >80% of the weight of the glycoprotein, N-glycosylation of Muc2 has been shown to be important for subsequent dimerization and maturation in the Golgi^24^. However, previous studies of Muc2 N-glycosylation were limited to in vitro cell studies where ER to Golgi transit was slower but maintained^24,25^. Cryo-EM and glycopeptide mass spectrometry studies have shown Muc2 C-terminal N-glycans influence the shape of the protein as these glycans line the dimerization domain^26^. Improper glycoprotein folding can prevent ER-Golgi progression, and while Muc2 is an abundantly synthesized intestinal glycoprotein, other glycoproteins could experience similar ER retention. Therefore, we measured ATF4, BiP, CHOP, and GRP78 as markers of ER stress. Each ER stress marker showed elevated expression in *benadrl* mice after DSS, with slightly increased DAB signal by immunohistochemistry in intestinal epithelial cells (Fig. 4a, b). The ER stress localizes specifically in the epithelial cells as demonstrated by immunohistochemistry of ATF4 and BiP in Mpi^ΔVillin^ mice (Fig. 4c). Similarly, Mpi^ΔVillin^ mice baseline expression of *AT4*, *BiP*, *and CHOP* were elevated (*p* = 0.032, 0.016, 0.0009, respectively) with only Grp78 trending upwards (Fig. 4d).

TUNEL staining revealed that 55% of ileal goblet cells were TUNEL positive in Mpi^ΔVillin^ mice as compared to 0% in Mpi^F/F^ (Fig. 4e, f). TUNEL staining increased in Muc2-negative enterocytes but to a much lower degree (5% Mpi^ΔVillin^ vs. 0% WT). In contrast, TUNEL staining was not increased in the colon of *benadrl* or Mpi^ΔVillin^ mice (Supplementary Fig. 3). These findings indicate that Mpi deficiency results in elevated ER stress and selective goblet cell apoptosis in the ileum.

### N-glycosylation regulates Muc2 O-glycosylation and goblet cell homeostasis

**Fig. 5:**
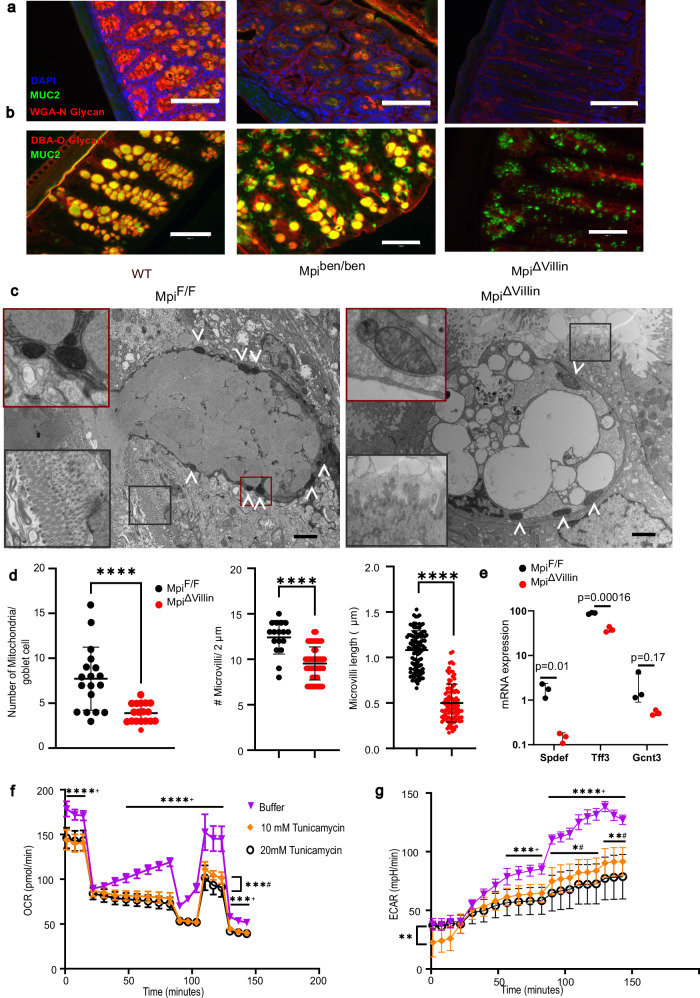
Mpi mutation affects glycosylation status that drives mucus synthesis and mitochondrial metabolism. **a** WGA and MUC2 immunofluorescent staining of Mpi^F/F^ and Mpi^F/F^; Mpi^ΔVillin^ colon tissues (scale bar = 75 μm). **b** DBA and MUC2 immunofluorescent staining of Mpi^+/+^, Mpi^ben/ben^, and Mpi^ΔVillin^ ileal tissues (scale bar = 50 μm). **c** Representative electron microscopy from Mpi^F/F^ and Mpi^ΔVillin^ goblet cell (*n* = 5 goblet cells each group from 2 different mice each group, Scale bar = 2 μm). **d** Quantitative analysis of mitochondria/goblet cell for Mpi^F/F^ vs Mpi^ΔVillin^ colons (*n* = 18 independent goblet cells from each group of mice), microvilli number counted across multiple 2 μm sections, and microvilli length measured (*n* = 2 Mpi^F/F^, 3 Mpi^ΔVillin^ mice per genotype). **e** Relative mRNA expression of goblet cell transcription factors—*Spdef, Tff3*, and *Gcnt3* (*n* = 3 colon samples from independent mice each group). **f**, **g** Given the reduced mitochondrial mass observed on EM, mitochondrial oxygen consumption rate (OCR) and extracellular acidification rate (ECAR) were measured in HT29-MTX cells (20,000 cells/ well) with deficient glycosylation (tunicamycin: 10 μg/mL- 10 T, 20 μg/mL- 20 T). (*n* = 8 replicates for Tris-HCl (0.08 M) 20 k, *n* = 16 replicates for 10 T 20 k and *n* = 14 replicates for 20 T 20 k. Results are the aggregate of a single experiment). Data expressed as means ± s.d. and significance was determined by: **d** two -tailed unpaired Student *t*-test, **e** Welch’s Test, or **f**, **g** Two-way ANOVA with Tukey’s multiple comparisons test. (*P < 0.05, **P < 0.01, ***P < 0.001 and ****P < 0.0001). **f**, **g** “+” indicates a significant difference between Buffer vs. 10 and 20 mM Tunicamycin, “#” indicates a significant difference between 10 vs. 20 mM Tunicamycin. Data is representative of at least 3 independent experiments. Spdef SAM Pointed Domain ETS Factor, Tff3 Serum trefoil factor 3, and Gcnt3 Glucosaminyl (N-acetyl) transferase 3, WGA Wheat Germ Agglutinin, MUC2 mucin 2, DBA Dolichos Biflorus Agglutinin.

Since we observed less Alcian blue staining in the colon, which indicated a lower level of glycosylation, we sought to dissect the effect of Mpi deficiency on the main secreted mucin, Muc2. We first confirmed that N-glycans were decreased in the *benadrl* hypomorphic mice and then absent in Mpi^ΔVillin^ mice by using the N-glycan-specific lectin wheat germ agglutinin (WGA) (Fig. 5a). Similarly, O-glycosylation, abundant on Muc2, is decreased in *benadrl* mice and absent in Mpi^ΔVillin^ mice (DBA stain, Dolichos Biflorus Agglutinin, Fig. 5b). Muc2 protein levels similarly appear decreased by immunohistochemistry, but it was unclear if this is due to less mucin hydration or surface area of the glycoprotein.

Ultrastructural analysis of goblet cells was performed by transmission electron microscopy to evaluate sub-cellular changes. The most striking difference in Mpi^ΔVillin^ colons as compared to Mpi^F/F^ mice was the large, vacuolated goblet cells with abnormal vesicles instead of orderly packages of mucus (Fig. 5c). These disorganized vesicles resemble lysosomes that have inadequately completed lysis. Additionally, the Mpi^ΔVillin^ goblet cells contained significantly (*p* ≤ 0.0001) fewer mitochondria as compared to Mpi^F/F^ mice (Fig. 5d), which could negatively affect the metabolic fitness. In enterocytes, the length and density of microvilli was significantly decreased (*p* < 0.0001, *p* < 0.0001, respectively), in Mpi^ΔVillin^ mice as compared to Mpi^F/F^ mice, which would affect the absorptive ability of the intestines (Fig. 4d). This goblet cell dysmorphology, mitochondrial loss, and shortened microvilli were also found in the ileum of Mpi^ΔVillin^ mice (Supplementary Fig. 4). The decreased number of goblet cells was assessed by detection of critical lineage factors such as *Spdef* (master regulator of goblet cell differentiation)*, Tff3* (goblet cell product for protection) and *Gcnt3* (mucin glycosylation) (Fig. 5e). Both the relative mRNA expression of *Spdef* and *Tff3* decreased significantly (*p* = 0.01, *p* = 0.00016, respectively) in Mpi^ΔVillin^ mice as compared to Mpi^F/F^ mice, which indicates Mpi and N-glycan deficiency is deleterious to goblet cell differentiation and secretory function.

Furthermore, mitochondrial respiration was functionally impacted in the HT-29 MTX goblet cell line when treated with the glycosylation inhibitor tunicamycin (inhibits the first step of N-linked glycosylation when UDP-GlcNAc is added to dolichyl phosphate)^27^ as determined by Seahorse assay (Fig. 5f). All three groups show decline in basal respiration, as expected as cells consume oxygen for energy production. Proton leak is stable across all groups during the experiment. However, there is a notable drop in ATP production in the tunicamycin-treated groups, and the maximal respiration capacity is decreased. Decreased spare respiratory capacity (difference between maximal and basal respiration) indicates a diminished ability to respond to increased energy demands like during stress. Furthermore, the decreased ECAR (extracellular acidification rate, Fig. 5g) suggests tunicamycin inhibits glycolysis. Dose-dependent effects of tunicamycin are observed for OCR during electron transport chain decoupling by FCCP (*p* < 0.001) and ECAR during oligomycin (*p* < 0.05), FCCP, and rotenone+ antimycin A treatments (*p* < 0.01). Overall, these findings demonstrate the diverse metabolic effects of loss of glycosylation via Mpi deficiency.

### Mpi deficiency supports pathogenic bacteria outgrowth

**Fig. 6:**
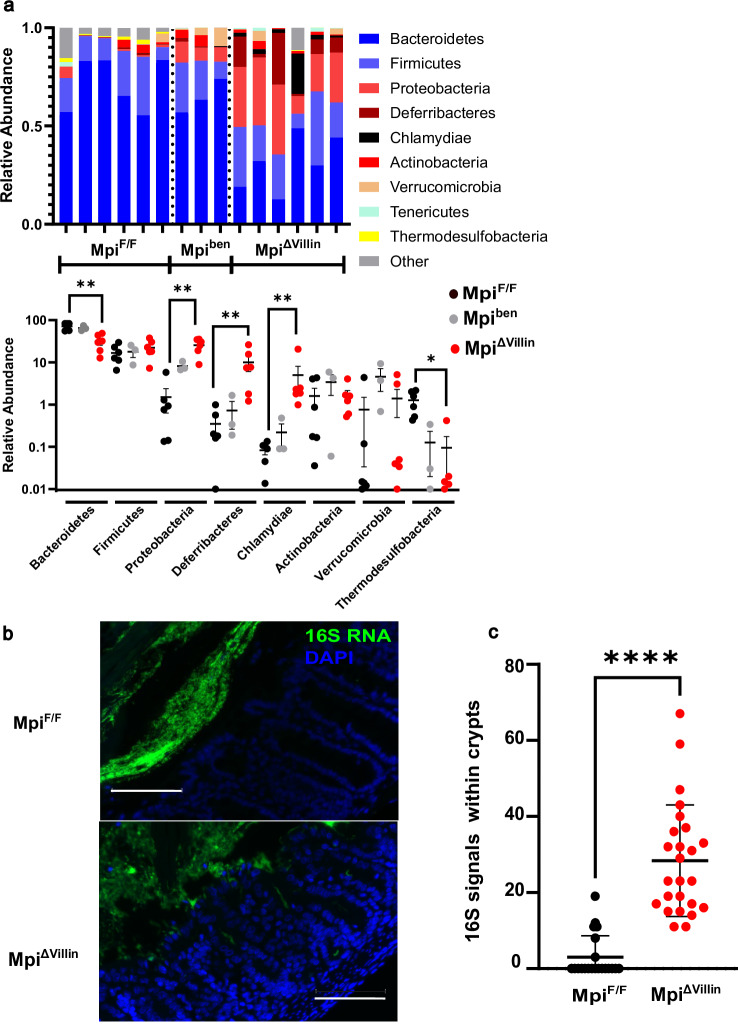
Dysbiosis and bacterial invasion. **a** Microbiome sequencing of Mpi^F/F^ (*n* = 6), Mpi^ben^ (*n* = 3) and Mpi^ΔVillin^ (*n* = 6); and quantitation of relative abundance below. (*P* value = 0.0238 for Bacteriodetes, Proteobacteria, Deferribacteres, Chlamydiae, and *P* value = 0.0119 for Thermodisulfobacteria). **b** Visualization of microbiota localization relative to the colon mucosal surface by 16S rRNA FISH (green) and DAPI (blue). Sections are representative of >10 littermates (*n* = 21 crypts from 3 Mpi^F/F^ mice, *n *= 25 crypts from 5 Mpi^ΔVillin^). Scale bars = 75 μm. Mice were co-housed littermates from intercrossed Mpi^ΔVillin^ mice. **c** Quantification of 16S+ signal beneath the crypt tip (*n* = 5 mice/genotype). **a**, **c** Data expressed as means ± standard deviation and significance was determined by two-sided Mann–Whitney *U*-test (**a**, non-parametric data) and two-sided Student *t*-test (**c**, parametric data). (*P < 0.05, **P < 0.01, ****P < 0.0001).

Muc2 is important for maintaining the barrier between intestinal epithelium and commensal bacteria. Furthermore, mucins provide nutrition to microbes to keep them in a well-balanced equilibrium with the host^28,29^. Comprising 80% of the weight of mucins, carbohydrate glycans are the nutritional source for commensal bacteria. Dysbiosis, an imbalance in the gut microbiome, can drive disease by bacteria penetrating the epithelial barrier or secretion of altered metabolites. In Mpi^ben^ mice, the intestinal mucus layer is decreased with a nearly complete loss in Mpi^ΔVillin^ colons^16^. 16S RNA sequencing of stools showed significant alterations in the Mpi^ΔVillin^ and Mpi^ben^ stools (Fig. 6a). The levels of Proteobacteria, Chlamydia, and Thermodesulfobacteria phyla in Mpi^ben^ mice were not significantly different from either Mpi^F/F^ or Mpi^ΔVillin^. The frequency of the symbiotic microbial phylum Bacteroidetes was 60% lower (*p* = 0.0036) in Mpi^ΔVillin^ compared to Mpi^F/F^. In contrast, bacteria such as Chlamydiae, Deferribacteres, and Proteobacteria were increased (*p* = 0.00899, *p* = 0.0058, *p* = 0.0019, respectively), contributing to a state unbalanced microbiome (Fig. 6a)^30^. These changes are more than just a shift in the natural microbiome abundance but rather an outgrowth of genera known to cause disease (*Chlamydia murinatum*). Accordingly, confocal imaging of 16S FISH probe hybridized bacteria found significant (*p* < 0.0001) bacterial penetration into colonic crypts of Mpi^ΔVillin^ when compared to Mpi^F/F^ colonic crypts (Fig. 6b).

### Mpi is essential for Paneth cell granulation

**Fig. 7:**
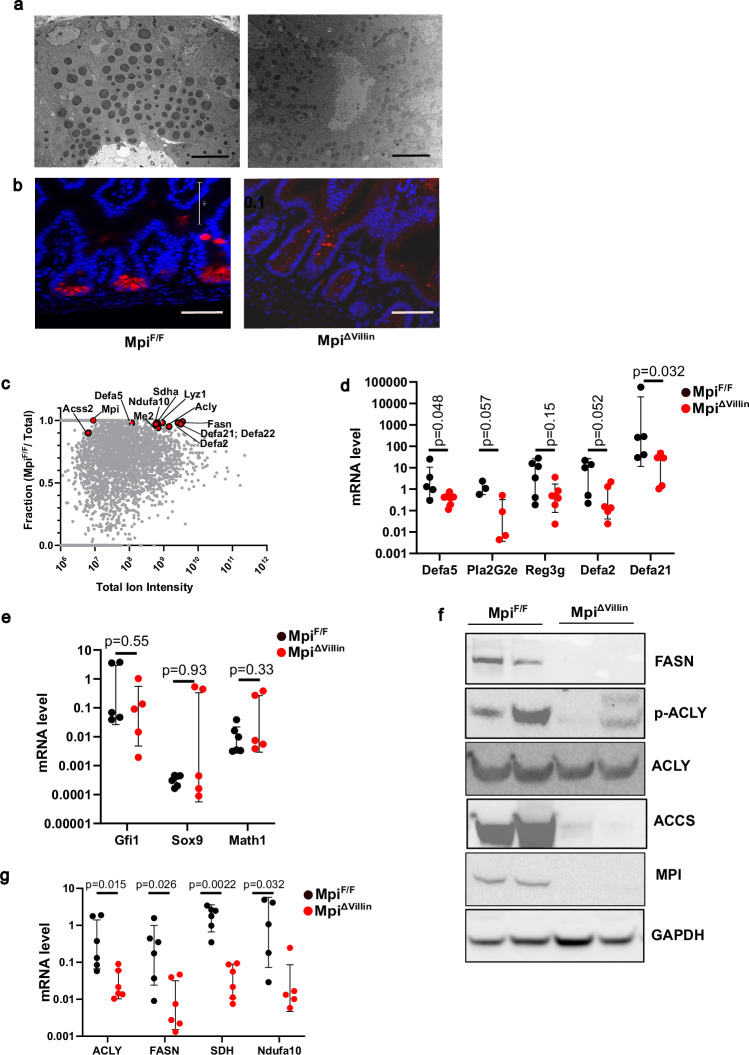
Paneth cell maturation and metabolic defectsa. Representative electron microscopy images from Mpi^F/F^ and Mpi^ΔVillin^ terminal ileum (*n* = 3 mice each group, scale bar = 8 μm). **b** UEA-1 immunofluorescent staining for Mpi^F/F^ and Mpi^ΔVillin^ tissue. UEA-1 Ulex Europaeus Agglutinin I (scale bar = 75 μm). **c** Composite plot of relative abundance as determined by mass spectrometry of Mpi^F/F^ and Mpi^ΔVillin^ terminal ileums (*n* = 1 for each genotype). Normalized proteins level [Mpi^ΔVillin^ / (Mpi^ΔVillin^ + Mpi^F/F^)] is plotted on the *Y*-axis vs Total Ion Intensity (Mpi^F/F^ + Mpi^ΔVillin^) on the *X*-axis. Points at *Y* = 1 denote proteins exclusively identified in the Mpi^ΔVillin^ sample; points at *Y* = 0 denote proteins exclusively identified in the Mpi^F/F^ sample. Protein abundance correlates with total ion intensity. **d** GAPDH normalized mRNA expression of Paneth cell specific genes*, Defa5 alpha defensin-5, Pla2G2e phospholipase A2 group IIE, Reg3g regenerating islet-derived protein 3 gamma, Defa-4 alpha defensin- 4* and *Defa21 alpha defensin- 21* (*n* = 5 and *n* = 7 for *Defa5*, *n* = 3 and *n* = 4 for *Pla2G2e*, *n* = 6 and *n* = 7 for *Reg3g*, *n* = 5 and *n* = 6 for *Defa4* and *n* = 5 for *Defa21* each independent ileal samples from Mpi^F/F^ and Mpi^ΔVillin^; *P < 0.05). **e** GAPDH normalized mRNA expression of Paneth cell specific genes, *Gfi1 Growth Factor Independent 1*, *Sox9* *SRY-Box Transcription Factor 9*, and *Math1 mouse atonal homolog 1* (*n* = 5 each for *Gfi1*, *n* = 6 and *n* = 5 for *Sox9*, and *n* = 6 and *n* = 5 for *Math1* independent ileal samples for Mpi^F/F^ and Mpi^ΔVillin^; * *P* < 0.05). **f** Immunoblot of FASN, p-ACLY, ACLY, ACCS, MPI, and GAPDH (*FASN Fatty Acid Synthase*, *ACLY* *ATP citrate lyase*, *ACCS Cytoplasmic Acetyl CoA Synthetase, MPI Mannose Phosphate Isomerase*, and *GAPDH glyceraldehyde-3-phosphate dehydrogenase*) in ileum lysates from 2 separate Mpi^F/F^ and Mpi^ΔVillin^ animals, representative image of 3 experiments. **g** GAPDH normalized mRNA expression of metabolic specific genes, *ACLY* *ATP citrate lyase*, *FASN Fatty Acid Synthase*, *SDH Succinate dehydrogenase*, and *Ndufa10* *NADH dehydrogenase [ubiquinone] 1 alpha subcomplex subunit 10* (*n* = 6 independent ileal samples each from Mpi^F/F^ and Mpi^ΔVillin^ for *ACLY*, *FASN*, *SDH*, and *n* = 5 independent ileal samples each from Mpi^F/F^ and Mpi^ΔVillin^ for *Ndufa10*). Data expressed as mean ± standard deviation and statistical significance was determined by Mann–Whitney test for (**d**, **e**, and **g**).

Ultrastructural analysis of the of the ileum revealed loss of Paneth cell dense core granules of Mpi^ΔVillin^ compared to the Mpi^F/F^ (Fig. 7a). Furthermore, similar to the colonic electron microscopy images, ileum microvilli density decreased, and goblet cells had a similar morphology (Supplementary Fig. 4). To confirm loss of Paneth cell granules, UEA-1 lectin staining was performed and confirmed loss of signal at the base of ileal villi. UEA-1 binds to fucosylated glycoproteins, and less staining in the Mpi^ΔVillin^ intestines is consistent with a loss of glycosylation due to *Mpi* deletion (Fig. 7b).

Paneth cell dense granules contain a mixture of anti-microbial peptides (defensins), lysozyme, and other defensive molecules. Paneth cells are found in the entire small intestine but are most dense in the terminal ileum, so we focused our analysis on the ileum^31^. Proteomic analysis of ileum lysates revealed *a* > 95% decrease in alpha defensin 5, 20, 22, lysozyme 1, and phospholipase A2 (Fig. 7c and Supplementary Table 3). We also found decreased expression of defensins 5 and 21 (*p* < 0.05) and nearly significant decreases in Pla2g2e and Defa2 expression (*p* = 0.057, 0.052, respectively), by using qPCR analysis (Fig. 7d), which complements the ultrastructural and mass spectrometry results for Mpi^ΔVillin^.

Next, we examined the gene expression of factors important for differentiation of Paneth cells and the secretory progenitors, using the ileum tissue lysates. Gfi1, Sox9 and Math1/Atoh1were unchanged in the Mpi^ΔVillin^ compared to the Mpi^F/F^ mice (Fig. 7e). This may represent a drive to create more Paneth cells since the ones present are not producing the necessary defensive molecules (Fig. 7d). Overall, these results indicate that Mpi loss not only affects the glycosylated, secretory granule contents but also Paneth cell maturation.

### Mpi deficiency reduces intestinal lipid metabolism

In addition to the loss of small anti-microbial peptides, mass spectrometry of ileal tissue lysates suggests several metabolic proteins shifted. Many of the altered proteins are enzymes involved in acetyl-CoA metabolism and fatty acid de novo synthesis, as well as components of mitochondrial metabolism such as NADH-ubiquinone oxidoreductase (Ndufa10), Succinate dehydrogenase (SDH), and malate/glyceraldehyde via pyruvate (malate enzyme, triose kinase). Furthermore, enzymes essential for converting Acetyl-CoA to fatty acids (Acly, Acc1, Fasn) in Mpi deficient lysates were decreased to <10% of the total intensity readings for each protein (Mpi^F/F^ + Mpi^ΔVillin^ protein) (Fig. 7g*)*. Proteomic findings were confirmed by western blot of Acc, Fasn, and Acly1 in Mpi^ΔVilliln^ mice (Fig. 7f). Repeating mass spectrometry on two more replicates (Supplementary Fig. 5a) also showed that fatty acid metabolic (Accs2, Acly, and Fasn), mitochondrial (Sdha and Ndufa10), and defensin proteins (Defa2, Defa20, Defa5) in Mpi ileal lysates were again decreased to <10% of the total protein intensity (Mpi^F/F^ + Mpi^ΔVillin^). Fatty acid enzyme loss was regulated at the transcriptional level as determined by RT-qPCR analysis. Gene expression of *Acly* and *Fasn* were decreased compared to wild type mice (Fig. 7g). A common feature of these metabolic genes is that they are all transcriptionally regulated by SREBP1. MPI-deficient LS174T cells demonstrated decreased p-ACLY and FASN (Supplementary Fig. 5b). Additionally, a total fatty acid profiling of ileal tissue showed FA16:0 (palmitic acid) dominated in the samples (Supplementary Fig. 5c–e). *Mpi*-deficient tissues trended lower or undetectable for FA16:0 and other fatty acids, but the results were not statistically significant.

## Discussion

This work addresses long-standing questions concerning N-glycan function for mucins in vivo. As mucins are 80% O-glycans by weight, defects affecting these post-translational modifications were a primary focus of research^32^. However, we have discovered that N-glycans are a prerequisite for Muc2 maturation, as genetic deficiency of *Mpi* causing N-glycan loss prevents any O-glycosylation. Using Alcian blue staining as a surrogate for Muc2 maturation, mutations affecting core 1- or core 3- O-glycans (C1galt1^f/f^; Villin^Cre^, C3Gnt^−/−^) or Golgi transport (*Trvp23b*, *Yipf3*) still retain some Alcian blue staining. However, when Mpi is deleted, there is no Alcian blue staining left, which resembles a Muc2^−/−^ strain or complete loss of O-glycans (double knockout of C1galt1^f/f^; Villin^Cre^ and C3Gnt^−/−^)^19,21,33^. This report provides mechanistic insight behind congenital diarrhea and PLE observed in human patients with MPI-CDG. By recognizing this mechanism, we highlight potential therapeutic opportunities to support gut health by augmenting gut N-glycans.

We analyzed the intestinal phenotypes using a targeted SNP variant (Mpi^benadrl^) and intestinal epithelial cell-specific deletion (Mpi^ΔVillin^)^16^. We demonstrated that loss of N-linked glycosylation from *Mpi* deficiency creates mice susceptible to DSS-induced colitis, dysbiosis, and loss of secretory goblet and Paneth cells. Mpi is of particular importance to intestinal homeostasis through several mechanisms, including loss of mucus barrier, anti-microbial peptides, ER stress, and lipid metabolism. The primary defect of mucus loss is the strongest observed from our collection of ENU-mutagenized DSS-susceptible phenotypes (*Tvp23b, Yipf6, Muc2*^*Winnie*^, Myo1d)^19,20,33–35^ and is comparable to Muc2 knockout^23^. While a previous study attributed congenital diarrhea in CDGs to heparin sulfate deficiency^36^, these data indicate *Mpi* and Muc2 N-glycosylation are critical for intestinal defense and homeostasis.

Although we could detect residual Muc2 in Mpi^ΔVillin^ colons by IHC, no O-glycans were detectable by DBA-lectin staining nor N-glycans in epithelial cells by WGA-lectin staining. Therefore, we found low levels of Mpi attenuates O-glycosylation, but deletion of Mpi prevents any O-glycosylation formation in the intestinal epithelial cells. Muc2 is attenuated in the *Mpi* hypomorphic allele and is nearly absent by IHC in the Mpi^∆Villin^ mice. O-glycans on secreted mucins such as MUC5B can be potent blockers of bacterial quorum sensing to prevent infection by pathogenic bacteria^37,38^. Muc2 glycans potently promote bacterial diversity, perhaps due to shorter, less extended glycans compared to MUC5B and Muc5ac^39,40^. Interestingly, the use of a high sugar diet changed the mucus thickness due to outgrowth of a mucus degrading bacteria^41^. The major functional studies were performed in vitro showing a slowed ER-Golgi transit time and Muc2 dimerization^24^. More recent work by the Gunner group has employed glycopeptide mass spectrometry and cryo-EM to map the C-terminal N-glycan sites^42^. These studies showed C-terminal N-glycans regulate Muc2 dimer shape and flexibility. The mild Muc2 phenotypes observed from these in vitro studies, significantly underestimate the profound loss of mucus, which we found. The significance of these findings has never been evaluated in vivo due to a lack of viable glycosylation-deficient models.

We shift the focus of glycosylation canon to N-glycans for their essential role in Muc2 maturation, subsequent O-glycosylation, and function in barrier defense. No in vivo study of N-glycans was previously possible due to no viable mouse model of N-glycan deficiency^43^. Previous attempts to create *Mpi* mutants resulted in embryonic lethality or no observable phenotype^11,43–45^. *Pmm2* mutants had significantly reduced viability and have not been examined for intestinal phenotype; emerging conditional knockout models may allow for this type of study^46^. Several CDG Type II mouse models have been created; they are viable but affect the quality (arrangement of carbohydrates) rather than an N-glycan knockout^47^. We use the term N-glycan KO because our previous work showed *Mpi*-deficient mice exhibited N-glycosylation sites that lacked attached carbohydrates(by glycopeptide analysis)^16^. This previous finding is supported by clinical studies of CDG Type 1 and is impactful by affecting N-glycans broadly^48^.

The ultrastructural changes of goblet cells showed highly disordered, shrunken, and aberrant structures that could be secretory vesicles or lysosomes. Furthermore, we found fewer mitochondria. This data was corroborated by mass spectrometry data with lower number of electron transport chain proteins. Lastly, electron microscopy demonstrated fewer intestinal villous projections, which can affect absorption of nutrients. Energy balance has been shown to be essential for intestinal homeostasis from creatine kinase-deficient mouse models^49^. Specifically, mitochondrial disruption can induce susceptibility to DSS colitis^20,21^.

We correlated the numerical mitochondrial defect to a functional impact on metabolism by Seahorse assay in a colonic goblet cell line (HT-29MTX). Combining the ECAR and OCR data provides a more complete picture of the metabolic changes induced by tunicamycin. The decrease in both glycolytic activity and mitochondrial respiration could come from a smaller mitochondrial mass, induced cell stress responses to preserve mitochondria, or improperly folded proteins involved in oxidative phosphorylation^22–24^. Glycosylation could impact mitochondrial proteins due to changes in protein folding and stability^25^ or protein targeting^26^. Most intracellular proteins are not glycosylated, so the impact of glycosylation on mitochondrial function may be more indirect.

While examining goblet cell ultrastructural morphology in the ileum, we noticed a striking decrease in Paneth cell dense granules. Unlike goblet cells, which secrete mucus, Paneth cells secrete anti-microbial peptides stored in dense granules at the base of small intestine crypts. Changes in the balance of Proteobacteria can represent a state of dysbiosis^28^. These microbes are normally increased in the small intestine, and perhaps the dual deficiency in Goblet and Paneth cells could explain the profound expansion of Proteobacteria in *Mpi* deficient mice^28,31^. This aligns with another recent report where Paneth cell lysozymes are decreased in an O-glycans defect secondary to *Tvp23b* KO. This was unexpected, because anti-microbial peptides are neither N- nor O-glycosylated.

Beyond protein secretory pathway defects, we observed defects in mitochondria and lipid metabolism that could be attributable to defective surface glycoprotein receptors. Since many surface receptors are glycosylated, lack of glycosylation could impact their folding, stability, transport, or ligand binding properties. This would make sense why intracellular pathways containing mostly non-glycosylated metabolic enzymes are affected. For example, EGFR, a critical epithelial growth factor receptor, has 12 N-glycan sites described, and in vitro studies removing these sites shows this inactivates its function^50^. Hepatic dyslipidemia has been found secondary to DSS-induced microbe crossing of the barrier^51^, however our observations were made in the intestines. Similarly, lipid metabolism is broadly affected with decreases in multiple enzymes. A common feature of all decreased lipid enzymes is that they are all regulated by SREBP proteins. SREBP exits the ER to the Golgi complexed with its escort protein, SCAP. While SREBP is not glycosylated, there are 3 predicted N-glycan sites on SCAP. A previous report demonstrates N-glycosylation stabilizes SCAP by reducing its interaction with Insig-1 to facilitate SCAP/SREBP trafficking from the ER to Golgi for activation^52^. Our report, therefore, identifies *Mpi* as a potential therapeutic target to modulate lipogenesis, which is important for metabolism and some malignancies.

We have separately reported that mannose supplementation reverses the low weight and other phenotypes of *Mpi* deficiency^16^. Mannose is found in fruits and berries and is absent in mouse chow diets, so is unlikely to influence the phenotype described. Mannose supplementation was recently linked to improving symptoms of DSS-induced colitis and IL-10 KO IBD models in mice^53^. A study by Dong et al. attributed the action of mannose to stabilizing lysosomes, but the link was not clear^53^. This could be a contributing factor but given the important role of glycosylation in Muc2 maturation, it is possible that mannose increases N-glycan precursors, supporting the synthesis of Muc2. Mpi, like many enzymes, could act as a rate-limiting step that could be bypassed by mannose^8,12,44^. If true, we would hypothesize mannose supplementation increases Muc2 production and protection from gut microbiome dysbiosis. Increasing gut protection from natural mucus could significantly aid several IBD disorders or other conditions affected by microbiome dysbiosis.

This study has opened several new questions on the role of N-glycans for functional Muc2. Through elegant studies of glycopeptide mass spectrometry and cryo-electron microscopy (cryo-EM)^42,54^ 17 occupied N-glycan sites have been mapped to the C-terminus of MUC2^54^. Enzymatic removal of these glycans creates a change in the dimer shape. However, the specific N-glycans responsible have not been identified. Other studies of glycosylated proteins, including the multimeric vWF or IgE, indicate specific sites may be essential for glycoprotein synthesis or secretion^55,56^. Therefore, we propose that the conserved oligomannose site is a critical N-glycosylation site required for Muc2 stability. Whereas disulfide bridges have been a focus of previous studies of MUC2 dimerization, this work highlights the importance of N-glycan post-translational modifications^37^. Determining the impact of specific N-glycan sites will provide interesting mechanistic insight into future studies.

This report addresses several long-standing questions in the fields of mucin biology, host defense, microbiome, and CDG. Our findings demonstrate the absolute necessity of N-glycans for intestinal mucin maturation, which is in turn critical for preventing pathogenic bacteria in the microbiome. Several CDG (including MPI-CDG) have been linked to decreased mucus production, but this study provides evidence that N-glycosylation is a necessary precondition before important O-glycans can be added.

## Methods

### Study approval

All animal experiments were performed in accordance with UT Southwestern IACUC-approved protocols (2021-103043).

#### Sex as a biological variable

We included equal numbers of male and female mice littermates. We initially evaluated whether sex contributed as a biological variable, but the strong autosomal nature of the mutation had the largest effect. Data from both sexes were aggregated for all subsequent experiments.

### Mice

A C57BL/6 male is mutagenized with N-ethyl-N-nitrosourea (ENU) and bred to produce G1 founder males^14,57^. G1 males are crossed with C57BL/6J females to create the G2 generation. Recessive mutations can be found in the G3 generation by a backcross of G2 females with their G1 father. A candidate *Mpi* mutation (p.H54R) was named *benadrl* and knocked-in as described elsewhere^16^. The *benadrl* allele was isolated by crossing C1 (1st generation CRISPR mice) to C57BL6/N mice, then intercrossing the C2 generation to test C3 mice with the homozygous *H54R* mutation. The Mpi-flox allele was created with flanking loxP sites around the 3rd *Mpi* exon as described elsewhere^16^. *Villin*-Cre (Vil1-Cre, JAX stock# 004586) was obtained from Jackson Labs. Mice 6–16 weeks of age were used in these studies. Mice are available upon request.

### Western blot

Tissues were lysed by sonication in NP-40 lysis buffer with protease phosphatase inhibitor cocktail (Sigma Aldrich, PPC1010), normalized to equivalent total protein levels (30 μg) using BCA Kit (Thermo Fisher Scientific, A55864) and separated by electrophoresis (Nupage 4–12% Tris gel, Invitrogen, NP0322BOX). Antibodies were used from Cell Signaling Technology to detect FASN, ACLY, ACC1, and AeCs, Thermo Fisher Scientific, and Abcam antibodies were used to detect MUC2, from tissue samples of Mpi^F/F^ and Mpi^ΔVillin^ (Supplementary Table 1).

### qPCR

Trizol® extraction of murine tissue in the Bioruptor Pico Sonication device (Diagenode) yielded pure total RNA used for qPCR experiments. qPCR was performed directly using the mRNA template by TaqPath™ 1-Step RT-qPCR Master Mix, CG (Thermo Fisher Scientific, A58666). Primers were purchased from IDT and 500 nM forward and reverse primers (Supplementary Table 2) were used with GAPDH Primetime qPCR primers (Mm.PT.39a.1, IDT) and SYBR green master mix to calculate normalized RNA expression. Expression data were normalized to GAPDH as described previously^11^.

### Hematology analysis

EDTA anti-coagulated blood was collected from the facial vein and measured for complete blood count by the HemaVet 950FS (Drew Scientific), which provided red blood cell count, size characteristics, and differential white blood cell count^16^.

### Histology

Tissues were fixed in 4% paraformaldehyde (ThermoScientific, AA47392-9M) or Carnoy’s fixative (if mucus thickness measured, Fisher Scientific, 18-601-490) for at least 48 h, then paraffin-embedded, sectioned at 5 μm, and stained by hematoxylin and eosin, or Alcian blue techniques by the histology core^13^.

### DSS treatment

2% DSS (Thermo Scientific, J63606-22) was used in drinking water for 7 days to induce colitis in Mpi^ben/ben^ mice as well as the wild-type mice 6–8 weeks old. Then the mice were sacrificed for collection of tissues and measuring the disease activity index^19^.

### Cell lines

The Lenti-CRISPR v2 (Addgene) vector along with 136 (targeting exon 2) and 1049 (targeting exon 7) guide RNA (Supplementary Table 2, Vector builder) were used to generate the LS174T-Mpi KO cell lines. Lentiviruses were produced with viral constructs, psPAX2 and pMD2G by co-transfection into Lenti-293T cells using polyethyleneimine (Fisher Scientific, AA4033136). The virus supernatant was collected 72 h after transfection and precipitated using PEG-it (System Biosciences Cat#LV810A1). Cells were centrifuged with virus supernatant in the presence of polybrene (Millipore Sigma Cat#TR1003G, 8 μg/ml) at 800 × *g* for 30 min and incubated for 3 days. For selection, cells were cultured in complete media containing puromycin (10 mg/mL, Fisher Bioreagents BP2956100) or blasticidin (10 mg/mL, Fisher Scientific, MT30100RB).

HT-29 MTx, LS174T, 136-7 vector only, 1049-7 vector only, and LS174T-Mpi KO cell lines (136-5 Mpi KO and 1049-5 Mpi KO) were cultured in DMEM media. The culture media for all the cells (HT-29, MTx, and LS174T) was supplemented with 10% FBS, 2 mM glutamine, 100 units/L streptomycin, 100 units/L penicillin. LS174T-Mpi KO cell lines were additionally supplemented with 10 mg/ml puromycin. Cells were maintained in 37 °C incubator with 5% CO_2_ and subcultured every 3 to 4 days in ratio of 1:3.

### Microbiome analysis

Two pellets of the stool samples were collected from Mpi^ben/ben^, Mpi^F/F^, and Mpi^ΔVillin^ into the tubes provided by Transnetyx and sent for the shotgun whole genome sequencing, as described previously^58^. The data were analyzed using the One-Codex platform^59^.

### Proteomics analysis of Ileum lysate

Ileum tissues from Mpi^F/F^ and Mpi^ΔVillin^ mice were collected and lysed. Samples were run just beyond the stacking gel (0.5 cm), stained with Coomassie blue, and then the entire stained bands were excised and sent in low retention Eppendorf tubes for mass spectrometry to the UTSW Proteomics Core^60^.

Samples underwent reduction and alkylation with TCEP and iodoacetamide (Sigma–Aldrich, I1149, C4706) and were subsequently digested overnight with trypsin (Pierce, 90057) using S-Trap micro columns (Protifi, K02MICRO10). Following peptide elution from the columns and solid-phase extraction cleanup with an Oasis HLB µelution plate (Waters, 186001828BA), the resulting peptides were reconstituted in 2% (v/v) acetonitrile (ACN) and 0.1% trifluoroacetic acid in water to a final concentration of ~0.5 μg/uL, based on an A205 reading by a NanoDrop (Thermo). Two microliters of each sample was injected onto an Orbitrap Fusion Lumos mass spectrometer (Thermo) coupled to an Ultimate 3000 RSLC-Nano liquid chromatography system (Thermo). Samples were injected onto a 75 μm i.d., 75-cm long EasySpray column (Thermo, PS-21548), and eluted with a gradient from 0 to 28% buffer B over 90 min. Buffer A contained 2% (v/v) ACN and 0.1% formic acid in water, and buffer B contained 80% (v/v) ACN, 10% (v/v) trifluoroethanol, and 0.1% formic acid in water. The mass spectrometer operated in positive ion mode with a source voltage of 2.5 kV and an ion transfer tube temperature of 300 °C. MS scans were acquired at 120,000 resolution in the Orbitrap and up to 10 MS/MS spectra were obtained in the Orbitrap for each full spectrum acquired using higher-energy collisional dissociation for ions with charges 2–7. Dynamic exclusion was set for 25 s after an ion was selected for fragmentation.

Raw MS data files were analyzed using MaxQuant v2.6.7.0 (https://maxquant.org), searched against the mouse protein database from UniProt (downloaded April 28, 2025, 63137 entries) with default parameters. The false-discovery rate cutoff was 1% for all peptides. Peptide peak intensities were used as peptide abundance values, and label-free quantitation was performed to compare protein levels across samples. Results were reported as total ion intensity, which is defined by MaxQuant as the sum of eXtracted Ion Current of all isotopic cluster for the identified amino acid sequence. Due to challenges with breeding to get enough Mpi^ΔVillin^ mice, only a pair of samples were initially analyzed. The same test was repeated on four mice and unfortunately, the protein levels were too different to combine results for statistical analysis. However, the results were compared qualitatively. Proteins within a similar functional pathway were selected for validation by qPCR or immunoblotting. The proteomics data served to guide these targeted validation experiments rather than to stand alone as evidence of differential protein abundance.

### Electron microscopy

Colon and ileal tissues were collected from Mpi^F/F^ and Mpi^ΔVillin^ mice then they were fixed in with 4% paraformaldehyde, 1.5% glutaraldehyde, and 0.02% picric acid in 0.1 M cacodylate buffer (created by the EM core) at 4 °C overnight before submission of the samples to the Electron Microscopy Core at UTSW. Tissues were imaged by the JOEL 1400+ microscope after being mounted on grids as described previously^19,49^.

### Immunohistochemistry

For IHC, sections were deparaffinized and rehydrated in xylene and gradation of ethanol solutions. This was followed by antigen retrieval using citrate buffer solution (Fisher Scientific, AAJ63950AK) at boiling temperature for 10 min. The primary antibodies incubated overnight at 4 °C, then incubated in secondary antibody tagged with horseradish peroxidase for 1 h at room temperature and visualized with DAB solution as described previously^19^. Images were taken using a EVOS Microscope.

### 16sRNA FISH

16s FISH was performed as described previously^19^. Briefly, colon tissues were fixed in Carnoy’s solution (Fisher Scientific, 18-601-490) for 3 days. Then the tissues were submitted to the Histo Pathology core for paraffin embedding and sectioning. Tissue sections were deparaffinized using two changes of xylene for 10 min each and then rehydrated using 95 and 90% ethanol for 10 min each followed by ddH2O for 10 min. Then the sections were incubated with fluorescently labeled 16 s RNA probes (Supplementary Table 2) in hybridization buffer consisting of 20 mM Tris·HCl (pH 7.4), 0.9 M NaCl, 0.1% SDS at 50 °C overnight in a humidified chamber^19,30^. The images were taken using an EVOS Microscope.

### Mitochondrial ETC activity measurements

An Agilent Seahorse XFe96 Analyzer was used for cellular oxygen consumption measurements of HT29-Mtx cells. HT29-Mtx cells were plated at 20,000 cells per well in 80 μL media and allowed to adhere overnight. The following day, cells were washed twice with 200 μL per well assay medium (DMEM (Sigma-Aldrich, D5030) with 10 mM glucose, 2 mM L-glutamine, 1 mM sodium pyruvate, and 1% penicillin/streptomycin), and 150 μL assay medium was added to each well after the second wash. Cells were transferred to a 37 °C, CO_2_-free incubator for 1 h. Standard calibration and baseline oxygen consumption measurements were performed using a 3-min “mix”/3-min “measure” cycle with three measurements recorded at baseline and after injection of each compound. The following inhibitor was used: 5 μg/mL Oligomycin, CCP, Antimycin A, and 20 μg/mL tunicamycin. Data collection was performed with WAVE (v.2.4.1.1) software^36^.

### Data reproducibility and statistical analysis

The mice used were from C57BL/6J background and of age 6–8 weeks old. No data were excluded, and investigators were not blinded. The unpaired Student’s *t*-test was used for comparisons between two parametrically distributed datasets or Mann–Whitney *U* test for two non-parametrically distributed datasets. GraphPad Prism 7 was used for analysis. *P* < 0.05 was considered significant. No pre-specified effect size; ≥3 mice per group were used.

### Reporting summary

Further information on research design is available in the Nature Portfolio Reporting Summary linked to this article.

## Supplementary information

Supplementary Information

Reporting Summary

Transparent Peer Review file

## Data Availability

Source data are available for the main article and the Supplementary Information files. Shotgun metagenomic sequencing data of stool bacteria has been uploaded to the NCBI database with Accession Numbers- SAMN50545625 [https://urldefense.com/v3/__https://www.ncbi.nlm.nih.gov/biosample/50545625__;!!MznTZTSvDXGV0Co!BQGPxYQeUK8bwOJGo1-fa3ubcsjPuKxxd-JfRApT_H7U6ZRoBuc7RM0edMYLRh0BuNoo0p4ToWmDMedqlmb0G7TDDwcjjE7K-1dMNUPScy0$], SAMN50545626 [https://urldefense.com/v3/__https://www.ncbi.nlm.nih.gov/biosample/50545626__;!!MznTZTSvDXGV0Co!BQGPxYQeUK8bwOJGo1-fa3ubcsjPuKxxd-JfRApT_H7U6ZRoBuc7RM0edMYLRh0BuNoo0p4ToWmDMedqlmb0G7TDDwcjjE7K-1dMf3kuv4s$], SAMN50545627 [https://urldefense.com/v3/__https://www.ncbi.nlm.nih.gov/biosample/50545627__;!!MznTZTSvDXGV0Co!BQGPxYQeUK8bwOJGo1-fa3ubcsjPuKxxd-JfRApT_H7U6ZRoBuc7RM0edMYLRh0BuNoo0p4ToWmDMedqlmb0G7TDDwcjjE7K-1dMHnw2Too$], SAMN50545628 [https://urldefense.com/v3/__https://www.ncbi.nlm.nih.gov/biosample/50545628__;!!MznTZTSvDXGV0Co!BQGPxYQeUK8bwOJGo1-fa3ubcsjPuKxxd-JfRApT_H7U6ZRoBuc7RM0edMYLRh0BuNoo0p4ToWmDMedqlmb0G7TDDwcjjE7K-1dM4CAkT2M$], SAMN50545629 [https://urldefense.com/v3/__https://www.ncbi.nlm.nih.gov/biosample/50545629__;!!MznTZTSvDXGV0Co!BQGPxYQeUK8bwOJGo1-fa3ubcsjPuKxxd-JfRApT_H7U6ZRoBuc7RM0edMYLRh0BuNoo0p4ToWmDMedqlmb0G7TDDwcjjE7K-1dMR471fyE$], SAMN50545630 [https://urldefense.com/v3/__https://www.ncbi.nlm.nih.gov/biosample/50545630__;!!MznTZTSvDXGV0Co!BQGPxYQeUK8bwOJGo1-fa3ubcsjPuKxxd-JfRApT_H7U6ZRoBuc7RM0edMYLRh0BuNoo0p4ToWmDMedqlmb0G7TDDwcjjE7K-1dMAeX_qsA$], SAMN50545631 [https://urldefense.com/v3/__https://www.ncbi.nlm.nih.gov/biosample/50545631__;!!MznTZTSvDXGV0Co!BQGPxYQeUK8bwOJGo1-fa3ubcsjPuKxxd-JfRApT_H7U6ZRoBuc7RM0edMYLRh0BuNoo0p4ToWmDMedqlmb0G7TDDwcjjE7K-1dM1S71kcg$], SAMN50545632 [https://urldefense.com/v3/__https://www.ncbi.nlm.nih.gov/biosample/50545632__;!!MznTZTSvDXGV0Co!BQGPxYQeUK8bwOJGo1-fa3ubcsjPuKxxd-JfRApT_H7U6ZRoBuc7RM0edMYLRh0BuNoo0p4ToWmDMedqlmb0G7TDDwcjjE7K-1dMkqzYeX8$], SAMN50545633 [https://urldefense.com/v3/__https://www.ncbi.nlm.nih.gov/biosample/50545633__;!!MznTZTSvDXGV0Co!BQGPxYQeUK8bwOJGo1-fa3ubcsjPuKxxd-JfRApT_H7U6ZRoBuc7RM0edMYLRh0BuNoo0p4ToWmDMedqlmb0G7TDDwcjjE7K-1dM-0USJd4$]. Proteomic data is accessible at the MassIVE repository (massive.ucsd.edu) Accession No: MSV000100002 [https://massive.ucsd.edu/ProteoSAFe/dataset.jsp?task=fa3f27fa925b46fdad8b8ec6ca55781d].
