# Supplementary material for: Mpi-driven N-glycosylation orchestrates mucin O-glycosylation and intestinal homeostasis
Source: Nat Commun. 2026 May 18;17:6548. doi: 10.1038/s41467-026-73100-5 (PMC13381762; doi:10.1038/s41467-026-73100-5)

Supplemental Material and Methods for:

Mpi-Driven N-glycosylation Orchestrates Mucin O-glycosylation and Intestinal Homeostasis

Authors: Avishek Roy, Steve Meregini, Hye-Jeong Cho, Zhenglan Chen, Aariz Zaki, Tandav Argula, Bruce Beutler, Jeffrey A SoRelle

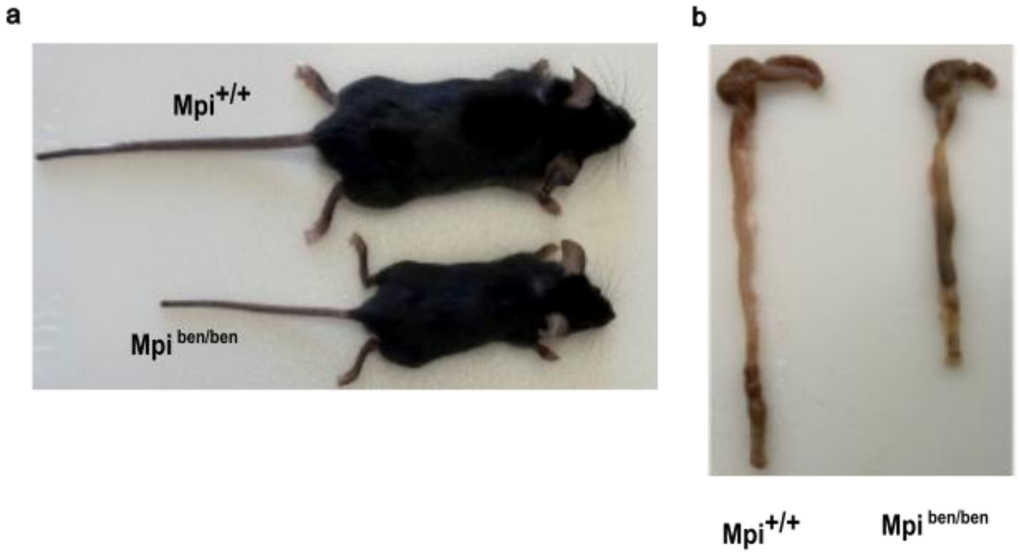

Supplemental Figure 1. Pictures of the mice and colons from the *benadryl* strain.

a ENU-mutagenized $Mpi^{ben/ben}$ mice were visibly smaller. **b** Representative colon morphology after 7 days of DSS treatment for $Mpi^{+/+}$ and $Mpi^{ben/ben}$.

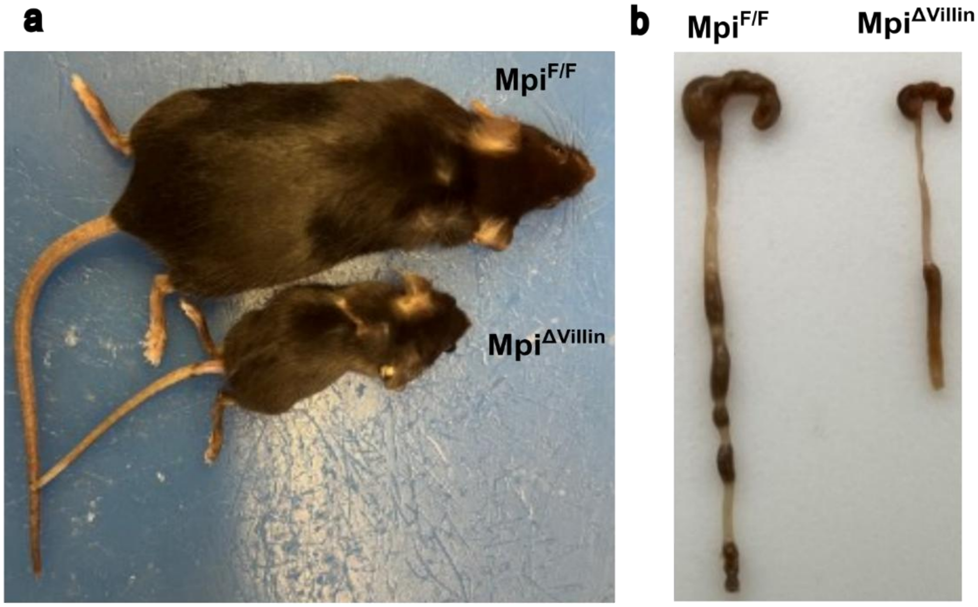

Supplemental Figure 2. Pictures of the mice and colons from the *Mpi^{flox}; Villin^{Cre}* strain. **a** Representative image for Mpi$^{F/F}$ vs Mpi$^{\Delta Villin}$ 4 week old mice. **b** Representative image of colons from Mpi$^{F/F}$ vs Mpi$^{\Delta Villin}$ 4 week old mice.

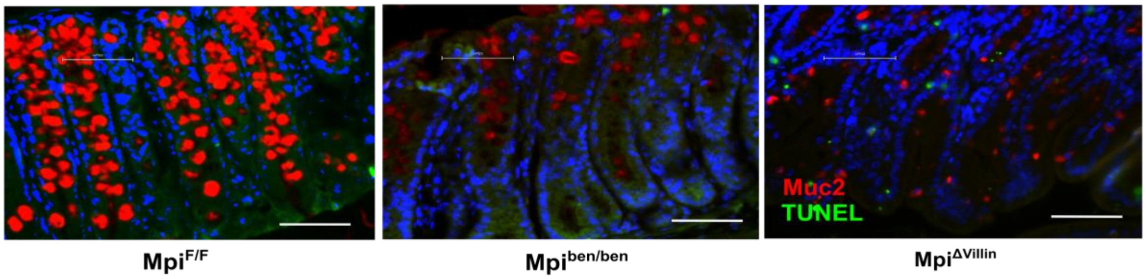

Supplemental Figure 3

Immunofluorescence of TUNEL (green) and Muc2 (red) from $Mpi^{F/F}$, $Mpi^{ben/ben}$ and $Mpi^{\Delta Villin}$ colon (n=3 mice each). Scale bar = 50μm.

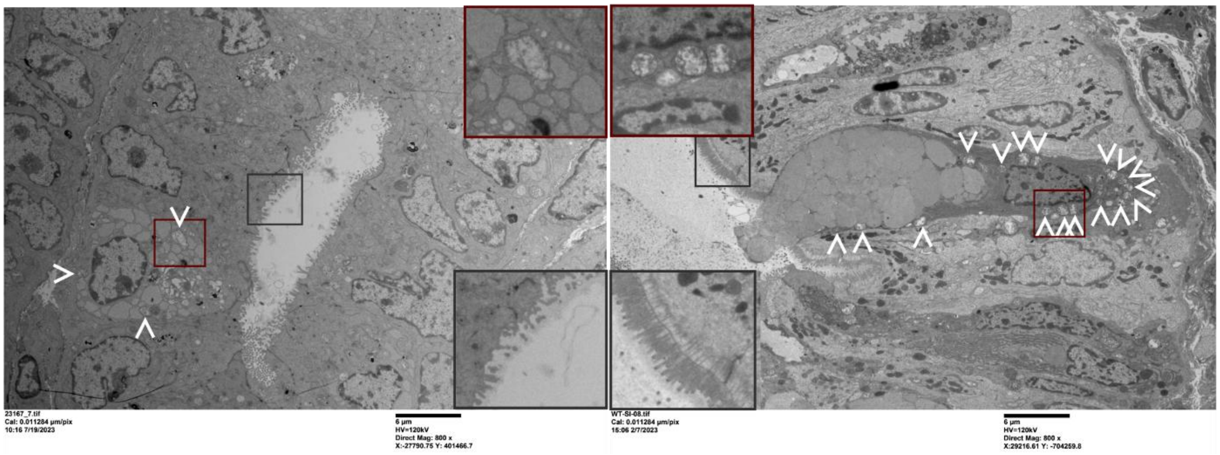

Supplemental Figure 4

Mpi deficient ileal ultrastructural changes. $Mpi^{f/f}$; $Villin^{Cre}$ epithelial cells of the ileum are shown (left) compared to wild type cells (right). The principle differences are goblet cell disorganized secretory vesicles, fewer goblet cell mitochondria (arrowheads, focus on red outlined box), and fewer and shorter brush border microvilli of the epithelial cells (focus in black outlined box).

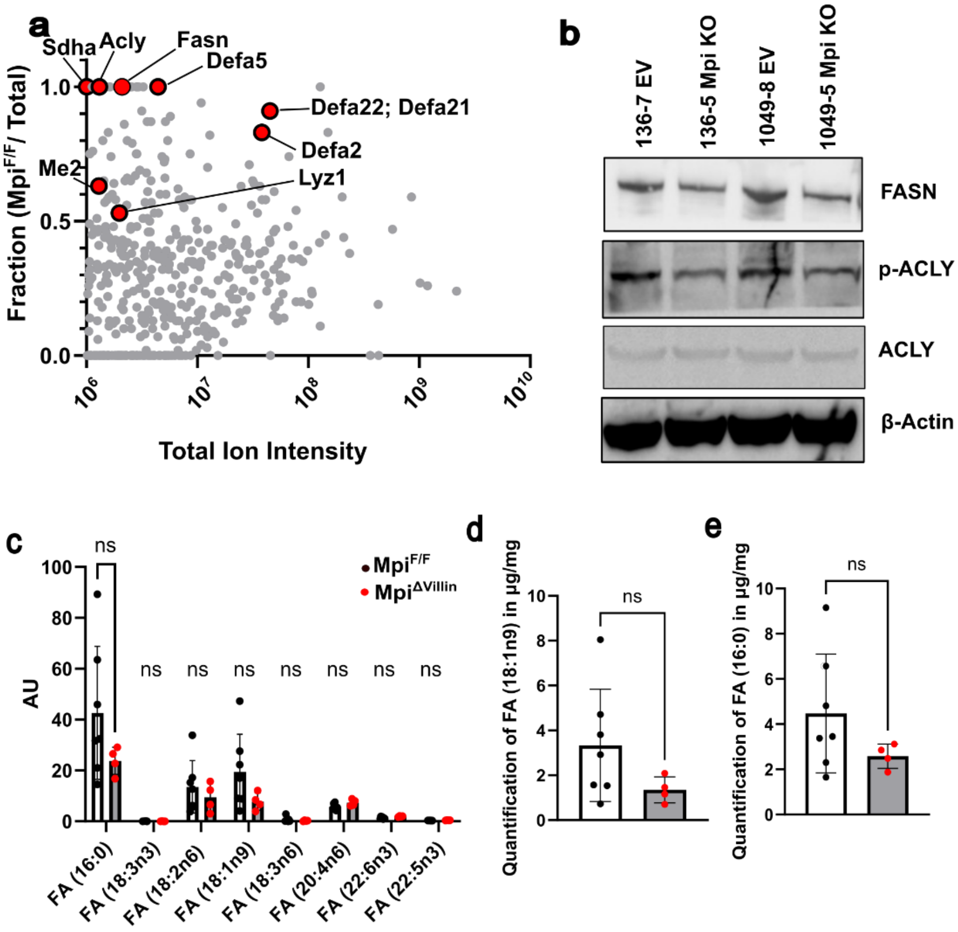

Supplemental Figure 5. Fatty Acid profiling and Mass spectroscopy replication

a Composite plot of relative abundance as determined by mass spectrometry of $Mpi^{F/F}$ and $Mpi^{\Delta Villin}$ terminal ileums (n = 2 for each genotype, Replication data). Normalized proteins level [$Mpi^{F/F}$/ ($Mpi^{\Delta Villin}$ + $Mpi^{F/F}$)] is plotted on the Y-axis vs Total Ion Intensity ($Mpi^{F/F}$ + $Mpi^{\Delta Villin}$) on the X-axis. Points at Y = 1 denote proteins exclusively identified in the $Mpi^{F/F}$ sample; points at Y = 0 denote proteins exclusively identified in the $Mpi^{\Delta Villin}$sample. Protein abundance correlates with spectral count. **b** Immunoblot of FASN, p-ACLY, ACLY and β-Actin (*FASN= Fatty Acid Synthase*, *ACLY=ATP citrate lyase* and *β-Actin= beta Actin*) in LS174T WT and Mpi KO cells -136-7 EV, 136-5 Mpi KO, 1049-8 EV and 1049-5 Mpi KO (where EV= Empty Vector) representative image of 3 independent experiments. **c** Quantitative analysis for different families of fatty acids in ileal samples from $Mpi^{F/F}$ and $Mpi^{\Delta Villin}$ mice (n= 6 for $Mpi^{F/F}$ and n= 4 for $Mpi^{\Delta Villin}$). **d, e** Quantitative analysis of FA (16:0) and FA (18:1n9) fatty acids in ileal samples from $Mpi^{F/F}$ and $Mpi^{\Delta Villin}$ (n= 7 for $Mpi^{F/F}$ and n=4 for $Mpi^{\Delta Villin}$, single experiment). C, D and E Data expressed as means ± s.d. and significance was determined as unpaired Student t-test. ns = not significant.

Suppl.Table 1 : List of Antibodies

**REAGENTS or RESOURCE**	**SOURCE**	**IDENTIFIER**
**Antibodies**		
Rabbit Recombinant Monoclonal MUC2 (1:1000) for WB and (1:500) for IHC	Abcam	Cat #EPR23479-47<br>Lot-1009676-35
Rabbit ATF6 Polyclonal (1:500) for IHC	Proteintech	Cat# 24169-1-AP<br>Lot-10008046
BiP Mouse Monoclonal Antibody (1:2000) for WB and (1:500) for IHC	Proteintech	Cat# 66574-1-Ig<br>Lot-10007101
Wheat Germ Agglutinin (WGA), Rhodamine (RL-1022) (1:500) for IHC	Vector Laboratories	Cat# RL-1022-5<br>Lot-ZH1213
Dolichos Biflorus Agglutinin (DBA), Rhodamine (RL-1032-2) (1:500) for IHC	Vector Laboratories	Cat# RL-1032-2<br>Lot-ZI0124
Ulex Europaeus Agglutinin I (UEA I), Rhodamine (RL-1062-2) (5ug/ml) for IHC	Vector Laboratories	Cat# RL-1062-2<br>Lot-ZK0525
Rabbit Monoclonal Fatty acid synthase (C20G5) (1:750) for WB	Cell Signaling Technology	Cat# 3180T<br>Lot-7
Rabbit Phospho-ATP-Citrate Lyase (Ser455) (1:700) for WB	Cell Signaling Technology	Cat# 4331T<br>Lot-4
Rabbit ATP-Citrate Lyase (1:700) for WB	Cell Signaling Technology	Cat#4332S<br>Lot-2
Rabbit Monoclonal AceCS1, (used to validate Acss2 in mouse) (D19C6) (1:750)	Cell Signaling Technology	Cat# 3658T<br>Lot-3
Mouse Monoclonal Anti-β-Actin (C4) (1:1000) for WB	Santa Cruz Biotechnology	Cat# sc-47778<br>Lot-K0824
GAPDH Mouse McAb (1:20000) for WB	Proteintech	Cat# -60004-1-1g<br>Lot- 10013030

Suppl. Table 2 : List of Primers

**Primer Name**	**Sequence**
Spdef Fw	AAGGCAGCATCAGGAGCAATG
Spdef Rv	CTGTCAATGACGGGACACTG
Tff3 Fw	GGCTGCTGCTTTGACTC
Tff3 Rv	AGCCTGGACAGCTTCAA
Gcnt3 Fw	AGAGTTCCATCAACTGCTCAGG
Gcnt3 Rv	CATCCTAAGGTAGTCGGCCTC
IL-10 Fw	CAAGCCTTATCGGAAATG
IL-10 Rv	CATGGCCTTGTAGACACC
IL-12α (P35 subunit) Fw	GCCTTGGTAGCATCTATGAG
IL-12α (P35 subunit) Rv	TCGGCATTATGATTCAGAGA
TNF-α Fw	TGGCCTCCCTCTCATC
TNF-α Rv	GGCTGGCACCACTAGTT
EuB338I	/5Cy5/GC TGC CTC CCG TAG GAG T
EuB338II	/5Cy5/GC AGC CAC CCG TAG GTG T
EuB338III	/5Cy5/GC TGC CAC CCG TAG GTG T
hMPI[gRNA#1049](reverse)(Exon 7)	GCAAAGCAGCCGATATCACCGTTTTAGAGCTAGAAATAGCAAGTTAAAATAAGGCTAGTCCGTTATCAACTTGAAAAAGTGGCACCGAGTCGGTGC
hMPI[gRNA#136] (Exon 2)	CAGCGAAGTGGCGCGGCTGTGTTTTAGAGCTAGAAATAGCAAGTTAAAATAAGGCTAGTCCGTTATCAACTTGAAAAAGTGGCACCGAGTCGGTGC

Suppl. Table 3 : List of Ileal Proteins with Abundance and Function

Name of the Protein	Fraction of Abundance {Mutant/(WT+Mutant)} (Fig 7B)	Fraction of Abundance {Mutant/(WT+Mutant)} (Supp Fig 3A)	Functions
Defa22; Defa21	0.02	0.09	Defensins
Defa5	0.02	0.00	Defensins
Defa2	0.03	0.17	Defensins
Lyz1	0.02	0.47	Defensins
Reg3g	0.27	0.00	Defensins
Acly	0.05	0.00	Fatty Acid Synthesis
Acss2	0.10	Not found	Fatty Acid Synthesis
Fasn	0.01	0.00	Fatty Acid Synthesis
Sdha	0.03	0.00	Krebs cycle
Me2	0.06	0.33	Krebs cycle
Ndufa10	0.25	0.00	Mitochondrial function

Suppl. Table 4 : List of Chemicals, Cell lines and strain of mice

**Chemical, peptides and recombinant proteins**	**SOURCE**	**IDENTIFIER**
Dextran Sodium Sulfate (DSS)	Thermo Fischer Scientific	Cat# J63606-22
Fetal Bovine Serum (FBS)	Rockland Immunochemicals,Inc	Cat# FBS-02-0050
DMEM (Dulbecos Modification of Eagles Medium) [+] 4.5 g/L glucose [-] L-glutamine, sodium pyruvate	Corning®	Cat#15-017-CV
Pen/Strep Cytiva	Fisher Scientific	Cat#SV30010LR1
Puromycin Dihydrochloride	Fisher Scientific	Cat#BP2956100
Protease and Phosphatase Inhibitor Cocktail	Sigma-Aldrich	Cat# PPC1010
Sterile DMSO	Sigma-Aldrich	Cat#D2438-5X10ML
PBS 10X without calcium, magnesium	Cytiva	Cat#SH30258.01
TBST 20X, pH 7.5	Bioworld	Cat#40120065-3
Trans-Blot	Bio-Rad	Cat#1704270
**Experimental models: Cell lines**		
LS174T	ATCC CL187	
136-7 LS174T	This paper	
136-9 LS174T	This paper	
1049-8 LS174T	This paper	
1049-5 LS174T	This paper	
HT29-MTX	Sigma-Aldrich	Cat# 12940401
**Experimental models: Organisms/Strains**		
**$Mpi^{ben/ben}$**	Mutagenetix	
**C57BL/6J**	Jackson Laboratories	Cat#000664
**$Mpi^{F/F}$**	Mutagenetix	
**$Mpi^{\Delta Villin}$**	Mutagenetix	

Supplemental Methods:

Protocol for Total Fatty Acid Analysis on GC-MS

A modified Bligh-Deyer extraction was used to extract lipids from the sample. The extraction was performed by crashing the protein by adding 1 mL of methanol to 0.4 mL ileal lysate sample in a glass tube. The tube was vortexed for 5 seconds and then centrifuged at 3500 rpm for 5 minutes. The supernatant was pipetted into a new glass tube, and 1 mL of dichloromethane and 1 mL of water into each sample glass tube. The mixture was vortexed for 5 seconds and then centrifuged at 3500 rpm for 5 minutes. The organic phase (bottom phase) was collected using a Pasteur pipette to transfer into a new glass tube and then dried under N2 gas. The hydrolysis procedure was performed by adding 1 mL of 0.5 M potassium hydroxide solution prepared in methanol using serological pipette), and then spiked with 50 ng FA standards [FA (16:0{2H31}), FA (18:1{2H9}), FA (20:4{2H8}), and FA (22:6 ω 3{2H5})] using micropipette into each dried sample glass tube. The mixture was tightly capped, vortexed for 5 seconds, and then placed in 80° C heat block for 1 hour then let to cool to room temperature. The hydrolyzed FAs were extracted by adding 1 mL of dichloromethane and 1 mL of water into each sample glass tube with hydrolysis solution. The mixture was vortexed for 5 seconds and then centrifuged at 3500 rpm for 5 minutes. The organic phase (bottom phase) was collected using a Pasteur pipette to transfer into a new glass tube and then dried under N2 gas. The derivatization procedure was performed by adding 50 μL 1% triethylamine in acetone and 50 μL of 1% pentafluorobenzyl bromide in acetone into each dried sample extract glass tube. The sample tubes were tightly capped, vortexed for 5 seconds, and allowed to sit at room temperature for 25 minutes. Then using a micropipette, 400 μL iso-octane was added to each sample tube. The tube was vortexed for 5 seconds and the entire content was transferred to a GC vial using Pasteur pipette. The sample was analyzed using an Agilent 7890/5975C by electron capture negative ionization equipped with DB-5MS column (40 m x 0.18 mm; 0.18 μm film thickness) from Agilent. The carrier gas used was hydrogen with a flow rate of 1.6 mL/min. The injection port temperature was set at 300° C. The sample injection volume was 1 μL. The initial oven temperature was set at 150° C, then increased to 200° C at 25° C/min, and then an increase of 8° C/min until a temperature of 300° C was reached and held for 2.2 minutes for a total run time of 16.7 minutes. The FAs were analyzed in selected ion-monitoring mode. The data was normalized to the internal standards. FAs with carbon lengths of ≤18, 20, and 22 were normalized to FA (16:0{2H31}), FA (18:1{2H9}), FA (20:4{2H8}), and FA (22:6 ω 3{2H5}), respectively. Data was processed using Mass Hunter software by Agilent.

Unedited immunoblots for Supplementary Figure 5

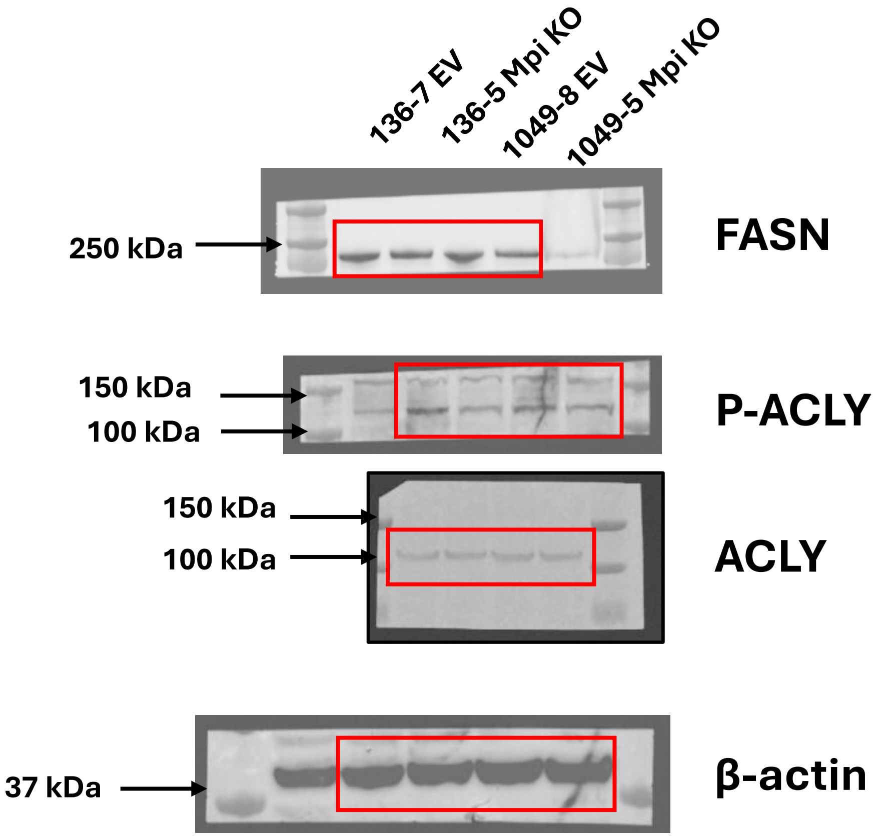

Supplement: Supplementary file 1 — Supplementary Information [file 41467_2026_73100_MOESM1_ESM.pdf]
