# Supplementary material for: Mpi-driven N-glycosylation orchestrates mucin O-glycosylation and intestinal homeostasis
Source: Nat Commun. 2026 May 18;17:6548. doi: 10.1038/s41467-026-73100-5 (PMC13381762; doi:10.1038/s41467-026-73100-5)
Supplement: Supplementary file 2 — Reporting Summary [file 41467_2026_73100_MOESM2_ESM.pdf]

nature portfolio

Corresponding author(s): SoRelle, Jeffrey

Last updated by author(s): 1/23/2026

Reporting Summary

Nature Portfolio wishes to improve the reproducibility of the work that we publish. This form provides structure for consistency and transparency in reporting. For further information on Nature Portfolio policies, see our Editorial Policies and the Editorial Policy Checklist.

Statistics

For all statistical analyses, confirm that the following items are present in the figure legend, table legend, main text, or Methods section.

n/a	Confirmed	
☐	☒	The exact sample size (*n*) for each experimental group/condition, given as a discrete number and unit of measurement
☐	☒	A statement on whether measurements were taken from distinct samples or whether the same sample was measured repeatedly
☐	☒	The statistical test(s) used AND whether they are one- or two-sided<br>*Only common tests should be described solely by name; describe more complex techniques in the Methods section.*
☒	☐	A description of all covariates tested
☒	☐	A description of any assumptions or corrections, such as tests of normality and adjustment for multiple comparisons
☐	☒	A full description of the statistical parameters including central tendency (e.g. means) or other basic estimates (e.g. regression coefficient) AND variation (e.g. standard deviation) or associated estimates of uncertainty (e.g. confidence intervals)
☐	☒	For null hypothesis testing, the test statistic (e.g. *F*, *t*, *r*) with confidence intervals, effect sizes, degrees of freedom and *P* value noted<br>*Give P values as exact values whenever suitable.*
☒	☐	For Bayesian analysis, information on the choice of priors and Markov chain Monte Carlo settings
☒	☐	For hierarchical and complex designs, identification of the appropriate level for tests and full reporting of outcomes
☒	☐	Estimates of effect sizes (e.g. Cohen's *d*, Pearson's *r*), indicating how they were calculated

Our web collection on statistics for biologists contains articles on many of the points above.

Software and code

Policy information about availability of computer code

Data collection	Not applicable
Data analysis	GraphPad Prism v.10.<br>For proteins extracted from ileum, protein identification was done using MaxQuant v2.6.7.0

For manuscripts utilizing custom algorithms or software that are central to the research but not yet described in published literature, software must be made available to editors and reviewers. We strongly encourage code deposition in a community repository (e.g. GitHub). See the Nature Portfolio guidelines for submitting code & software for further information.

Data

Policy information about availability of data

All manuscripts must include a data availability statement. This statement should provide the following information, where applicable:

- Accession codes, unique identifiers, or web links for publicly available datasets
- A description of any restrictions on data availability
- For clinical datasets or third party data, please ensure that the statement adheres to our policy

Shotgun metagenomic sequencing data of stool bacteria has been uploaded to the NCBI database with Accession Nos-SAMN50545625, SAMN50545626, SAMN50545627, SAMN50545628, SAMN50545629, SAMN50545630, SAMN50545631, SAMN50545632, SAMN50545633. Proteomic data is accessible at the MassIVE repository (massive.ucsd.edu) Accession No: MSV000100002.

Research involving human participants, their data, or biological material

Policy information about studies with human participants or human data. See also policy information about sex, gender (identity/presentation), and sexual orientation and race, ethnicity and racism.

Reporting on sex and gender	Not applicable
Reporting on race, ethnicity, or other socially relevant groupings	Not applicable
Population characteristics	Not applicable
Recruitment	Not applicable
Ethics oversight	Not applicable

Note that full information on the approval of the study protocol must also be provided in the manuscript.

Field-specific reporting

Please select the one below that is the best fit for your research. If you are not sure, read the appropriate sections before making your selection.

☒ Life sciences ☐ Behavioural & social sciences ☐ Ecological, evolutionary & environmental sciences

For a reference copy of the document with all sections, see nature.com/documents/nr-reporting-summary-flat.pdf

Life sciences study design

All studies must disclose on these points even when the disclosure is negative.

Sample size	No sample size calculation was performed. In general at least 4 mice were used. This sample size was sufficient to demonstrate statistically significant differences in comparisons between two unpaired experimental groups by unpaired t-test, Mann Whitney U test, or ANOVA, where indicated.
Data exclusions	None.
Replication	Repeated tests are specified in figure legends.
Randomization	Not relevant, age-matched littermate controls used with specified genotypes.
Blinding	Blinding was not possible as mutant mice were visibly different. Quantitation of goblet cells, electron microscopy, and 16S were performed by a co-author blinded to the source of the images.

Reporting for specific materials, systems and methods

We require information from authors about some types of materials, experimental systems and methods used in many studies. Here, indicate whether each material, system or method listed is relevant to your study. If you are not sure if a list item applies to your research, read the appropriate section before selecting a response.

Materials & experimental systems

n/a	Involved in the study	
☐	☒	Antibodies
☐	☒	Eukaryotic cell lines
☒	☐	Palaeontology and archaeology
☐	☒	Animals and other organisms
☒	☐	Clinical data
☒	☐	Dual use research of concern
☒	☐	Plants

Methods

n/a	Involved in the study	
☒	☐	ChIP-seq
☒	☐	Flow cytometry
☒	☐	MRI-based neuroimaging

Antibodies

Antibodies used	Rabbit Recombinant Monoclonal MUC2; Abcam ;Cat #EPR23479-47<br>Rabbit ATF6 Polyclonal; Proteintech; Cat# 24169-1-AP<br>BiP Mouse Monoclonal Antibody; Proteintech; Cat# 66574-1-Ig<br>Wheat Germ Agglutinin (WGA), Rhodamine (RL-1022); Vector Laboratories; RL-1022-5

	Dolichos Biflorus Agglutinin (DBA), Rhodamine (RL-1032-2); Vector Laboratories; RL-1032-2<br>Ulex Europaeus Agglutinin I (UEA I), Rhodamine (RL-1062-2); Vector Laboratories; RL-1062-2<br>Rabbit Monoclonal Fatty acid synthase (C20G5); Cell Signaling Technology; Cat# 3180T<br>Rabbit Phospho-ATP-Citrate Lyase (Ser455); Cell Signaling Technology; Cat# 4331T<br>Rabbit ATP-Citrate Lyase; Cell Signaling Technology; Cat#4332S<br>Rabbit Monoclonal AceCS1 (D19C6) Cell Signaling Technology Cat# 3658T<br>Mouse Monoclonal Anti-β-Actin (C4) Santa Cruz Biotechnology Cat# sc-47778
Validation	All primary antibodies used in this study were commercially purchased and validation was performed by the individual companies. Validation data for the specific application is present on the data sheets provided by the company websites

Eukaryotic cell lines

Policy information about cell lines and Sex and Gender in Research

Cell line source(s)	HT29-MTX (Sigma)<br>LS174T (ATCC)
Authentication	All cell lines were commercially purchased and the manufacturer tested and authenticated these cells, including morphological testing
Mycoplasma contamination	All cell lines were periodically tested for mycoplasma and all tested negative
Commonly misidentified lines<br>(See ICLAC register)	None used in this study

Animals and other research organisms

Policy information about studies involving animals; ARRIVE guidelines recommended for reporting animal research, and Sex and Gender in Research

Laboratory animals	Laboratory mice were on the C57BL/6 background. Strains are specified in the methods.
Wild animals	Not applicable
Reporting on sex	Age and sex matched mice from the same litters were used with specified genotypic controls.
Field-collected samples	Not applicableNot applicable
Ethics oversight	The University of Texas Southwestern IACUC approved the animal studies on this project.

Note that full information on the approval of the study protocol must also be provided in the manuscript.

Plants

Seed stocks	Not applicable
Novel plant genotypes	Not applicable
Authentication	Not applicable
