# Supplementary material for: Mpi-driven N-glycosylation orchestrates mucin O-glycosylation and intestinal homeostasis
Source: Nat Commun. 2026 May 18;17:6548. doi: 10.1038/s41467-026-73100-5 (PMC13381762; doi:10.1038/s41467-026-73100-5)
Supplement: Supplementary file 3 — Transparent Peer Review file [file 41467_2026_73100_MOESM3_ESM.pdf]

nature portfolio

Peer Review File

Mpi-Driven N-glycosylation Orchestrates Mucin O-glycosylation and Intestinal Homeostasis

Corresponding Author: Dr Jeffrey SoRelle

This file contains all reviewer reports in order by version, followed by all author rebuttals in order by version.

Version 0:

Reviewer comments:

Reviewer #1

(Remarks to the Author)
Key results
The manuscript by Roy and Sorelle et al. establishes that dampened mannose production (a surrogate for N-glycosylation), as observed in congenital disorders of glycosylation, can compromise intestinal homeostasis to the same extent as MUC2 knockout, a finding which elevates the importance of mucin N-glycosylation beyond the understood functions of ER transport and dimerization. The authors achieve this through a first-in-class murine model of mannose phosphate isomerase deficiency, with both general and intestine specific knockout strains being employed to confirm goblet cell reduction, severe colon pathology, elevated ER stress, limited mucin production, increased inflammation, and dysbiosis as a direct outcome of limited N-glycosylation. The murine strains presented thus represent both a step forward in understanding the role of N-glycosylation in production of protective MUC2 and antimicrobial peptides, and in providing a robust system by which to screen therapeutic options.

Taken together, this work represents a significant advance for the field, and we recommend publication provided that the following points are addressed.

Validity:
The data provided in figures 3B and 3D show elevated expression of specific ER stress response genes in their MPI knockout mice; however, given the established importance of N-glycosylation for general ER proteostasis and chaperone activity (1), can the authors confidently claim that this is due specifically to Muc2 accumulation/misfolding?
“Cryo EM and glycopeptide mass spectrometry studies have shown Muc2 C terminus N-glycans influence the shape of the protein as these glycans line the dimerization domain21. Improper glycoprotein folding can prevent ER-Golgi progression. Consequently, it appears Muc2 accumulates in the endoplasmic reticulum increasing ER stress markers of AT4, BiP, CHOP and Grp94 by mRNA expression in benadryl mice after DSS challenge (Fig 3B) and in MpiΔVillin at baseline (Fig 3D). ”

Significance:
We recommend the authors better connect the following four statements of significance above with their results, which were focused on a model of induced colitis starting from a point of CDG-MPI Mannose deficiency. We furthermore do not see how the data presented could establish N-glycosylation as more significant than O-glycosylation for “maintaining intestinal homeostasis.” The manuscript establishes mannose production (a surrogate for N-glycosylation) as essential to the production of mucin, a finding of great significance, but it is established in the literature that mucin attributes much of its microbe feeding and modulating activity, when formed, to the presence and distribution of specific O-glycan structures (2-6).

1. “While O-glycosylation makes up >80% of the weight of the glycoprotein, N-glycosylation has been shown to be important for subsequent dimerization and maturation in the Golgi.”
2. “Furthermore, mucins provide nutrition to microbes to keep them in a well- balanced equilibrium with the host. Comprising 80% of the weight of mucins, carbohydrate glycans are the nutritional source for commensal bacteria. Loss of Muc2 O-glycans secondary to N-glycan loss creates concern for dysbiosis. Dysbiosis, an imbalance in the gut microbiome, can drive disease by bacteria penetrating the epithelial barrier or secretion of altered metabolites. This work addresses long standing questions concerning N-glycan function for mucins in vivo. As mucins are 80% O-glycans by weight, defects affecting these post-translational modifications were thought to be more impactful. However, we have discovered that N- glycans are even more important in Muc2 maturation as genetic deficiency of Mpi causing N-glycan loss leads to a phenotype as strong as

Muc2 deletion."

3. "While immunohistochemistry can detect residual Muc2 in MpiΔVillin mice, remarkable lack of mucus barrier thickness, concomitant bacterial invasion, and dysbiosis. We found the residual Muc2 was no O-glycosylated, which is a mechanism for its gel-like properties that underly barrier functionality. Since >90% of glycans on Muc2 are O-glycans, the role of N-glycans has been understudied. "

4. "several congenital disorders of glycosylation (including MPI-CDG) and now has been linked to loss of mucin protection. Therefore, while mucins are heavily O-glycosylated, N-glycans have an essential role in maintaining intestinal homeostasis."

Data and methodology:
The quality and presentation of the data are sound.

Analytical approach:
We find the analysis of the presented data to be sound.

Clarity and context:
Two following statements in the results and discussion should be referenced if the prior work of the authors is used to make the argument.
1. "During an ENU-mutagenesis forward genetic screen, a mouse strain named benadryl associated with a visible and allergic phenotype (Fig. 1 A,B, p=1.6x10-12, recessive). This strain mapped to a missense variant p.H54R in the mannose phosphate isomerase gene named Mpi. We have previously described this strain, which is notable for recreating most features of the human congenital disorder of glycosylation MPI-CDG including congenital diarrhea…"
2. "We use the term N-glycan KO 397 because our previous work showed Mpi deficient mice had N- glycan sites without any carbohydrate at glycan sites (by glycopeptide analysis)."

The Introduction is difficult to understand and has poor syntax (see the specific examples below). The results and discussion sections present stronger arguments, but it is hard to defend the impact of this work without a well-written introduction to provide the necessary context.
Examples:
1. "The role of the mucins in the gut is not only restricted for their protective function but also impact the dynamics of the changing microbiome owing to the mucus-bacterial symbiotic relationship."
2. "One of the prominent O-glycosylated family of mucins lining gut epithelium is MUC2 and glycosylation status determines the gel-forming property of MUC2 in the form of mucus is essential for the gut protective barrier"

References
We recommend adding further references in the specific areas above. Below are the references that we are citing in this review:

1. Nat. Chem. Biol. 10, 902–910 (2014). https://doi.org/10.1038/nchembio.1651
2. Nat. Microbiol. 6, 574–583 (2021). https://doi.org/10.1038/s41564-021-00876-1
3. Nat Chem Biol 19, 778–789 (2023). https://doi.org/10.1038/s41589-023-01272-y
4. Cell Host Microbe 31, 1371-1385 (2023). https://doi.org/10.1016/j.chom.2023.07.003
5. Sci Rep 13, 13982 (2023). https://doi.org/10.1038/s41598-023-40497-8
6. Bergstrom, K., & Xia, L. (2022). Gut Microbes, 14.
https://doi.org/10.1080/19490976.2022.2052699

Reviewer #2

(Remarks to the Author)
The manuscript by Roy et al evaluated the role of the N-glycosylation enzyme Mpi in intestinal homeostasis. This work is relevant to congenital disorders caused by Mpi mutations and to understanding the role of N-glycosylation for gut health. The experiments are based on i) Mpi hypomorphic mice obtained by mutagenesis or ii) specific deletion (KO) of Mpi in intestinal epithelial cells. The results show that Mpi is crucial for mucus secretion and homeostasis of goblet and Paneth cells. Disruption of Mpi leads to dysbiosis and inflammation. These data are novel and expand the understanding of the importance of N-glycoslyation in the intestine and may lead to novel therapeutic strategies. The results seemed to be technically sound and state of the art methods were used.

However, the overall quality of the manuscript is suboptimal since there are many typos, some sections appear to have been copied and pasted from other documents, the order of the figure panels does not follow the description of the results, some experiments described in the materials and methods are not presented in the results (and vice versa) and the discussion critically lacks references to previous work. Moreover, the procedure used to generate the mouse models is not detailed in sufficient detail. All these important limitations of the manuscript can be easily corrected after a careful proofreading.

Previous studies using Mpi total KO mice (e.g. 10.1074/jbc.M511982200) are not mentioned in the manuscript and the lethal phenotype of the total KO should be presented to justify the choice to focus on a hypomorphic model. Knowing the phenotype of the total KO mice, the authors should provide the litter size and/or mortality rates in their hypomorphic and conditional KO mice.

The authors use the term "Benadryl strain" or "Benadryl allele" in the introduction and materials and methods to refer to the Mpi hypomorphic model. I understand that this is the designation chosen by the authors to name the phenotype of these mice but this is not clearly explained and this name may be misleading given the antihistamine effect of this drug, which is unrelated to the current study.

More experimental details should be provided regarding the generation of the transgenic mice. The choice of the candidate Mpi105 mutation (p.H54R) should be justified in the methods. I actually understood much better how this mutation was identified when I read the introduction of another publication by the authors (10.1038/s41467-023-39398) where their forward genetic screen is much more clearly explained. What are the functional consequences of the studied SNP mutation on Mpi activity? How did the authors exclude mutations other than in Mpi in the ben/ben mice?

The authors state that the mutation was knocked-in (Line 106) which is confusing since mutations were generated with ENU mutagenesis. The authors refer to an unpublished procedure (Lin et al, submitted supplement). There are also mention of "CRISPR Cas9 targeted mice", for instance in the legend of Figure 1. All these procedures should be clarified.

It is stated at the end of the introduction that "benadryl strain displayed reversal of intestinal physiological disorders simply by administration of Mannose" but no experimental data support this claim. This experiment with mannose supplementation would have indeed clearly reinforced the significance of the study by providing therapeutic perspectives but these data seems to have been removed from the manuscript or maybe presented elsewhere but without providing the reference. The mannose content of the diet fed to the mice should be reported since it may highly influence the phenotype of Mpi deficiency.

In the tissue specific mouse model of Mpi deletion, the authors use the term "intestine-specific N-glycan deficiency"/"intestinal specific KO", whereas the deletion of Mpi was performed specifically in the intestinal epithelium (Villin Cre mice). The manuscript should be corrected to clearly state that the knockout was restricted to the intestinal epithelium, while N-glycosylation remains functional in other intestinal cells. The authors state "to test whether the colitis phenotype was intrinsic to the colon, we used an intestinal epithelial cell specific knockout (Mpi flox/flox ; VillinCre : MpiΔVillin)" (Line 223). However, Villin is also expressed in the small intestinal epithelium and the phenotype cannot be attributed to the loss of Mpi in the colon only. There is also a confusion in the manuscript between villi (small intestine) and crypts (colon and small intestine), see for instance line 300 "colonic villi".

There is no information provided in this manuscript regarding the type of intestinal epithelial cells that express Mpi. This information could be retrived from scRNA-seq dataset and would help to understand the cellular mechanisms of the KO.

The gut microbiota composition was presented in the intestinal epithelium Mpi KO mice but not in ben/ben mice. These data should be provided to link Mpi activity with microbial changes.

The format of the reference is not the same in the introduction and materials and methods.
The materials and methods lack critical details (indicated below) and contains many typos and should be carefully corrected.

One example of the lack of care taken in drafting the manuscrit:
In legend of Figure 6 " Microbiome sequencing of Mpi F/F and MpiΔVillin (Update the labels to be consistent- you can mark them as 1, 2, 3 but put Mpi F/F below with a line to indicate they are in the same group".

There is no statistical analysis section. The number and age of mice used in each experiment is not provided in the methods section.

There is no data availability section (e.g. microbiome, proteomics) and supplementary materials are not provided.

The figure panels are not presented in the order of the description in the results which makes the reading much more difficult.

Some experiments related to LS174T cell line MPI KO are presented in the materials and methods section and in figure S1 but are not described in the results.

The discussion is not referring enough to references since previous results are mentioned by the authors but without citations.

Specific comments

69-71
"The presence of this post translational modification primarily in surface and secreted protein, which are involved in many aspects of physiology; hence, modulations in glycosylation may affect many physiologic and disease process" This sentence should be reworded.
71-72
It may be more relevant to focus on glycosylation defect-related diseases observed in the gut rather than immunoglobulin therapeutics or cardiovascular diseases.
75-76
There is no verb in this sentence.

90
It is not clear to what "Mpi phenotype" refers to
93
"wrt" should be corrected. This sentence is very long and should be reworded.
94
the "benadryl strain" was not introduced before
114
"phosphatase inhibitor cocktail (Company)" the company is not provided
118
All antibodies do not target MUC2
138
what was the exact concentration of DSS used? This can influence greatly the phenotype.
142
Details on the vectors should be provided. The culture of HT29-MTX cells is not described.
154
Typo example: "Tissues from Mpi F/F and MpiΔVillin mice were collected run on (Nupage 4-12% Tris gel) (Invitrogen) for 15 mins"
168
dilution of each antibody should be provided.
175
The 16S probe sequence is not provided. I guess the staining was performed on tissues fixed in Carnoy, which is not described.
183
The Figure 4C mention is not expected here.
200
A more specific wording than “visible” should be used. The allergy phenotype is not presented.
202
The reference where this strain was presented before should be mentioned.
207
the reference of the findings of your group should be provided.
"The strength of this phenotype most closely resembles Muc2 KO" a word is missing and a reference for the MUC2 KO should be provided.
215
"fewer goblet cells lead to less mucus and higher levels of neutrophils in Mpi ben/ben" This causality link was not demonstrated at this stage.
220
Why do we jump from figure 1 to figure 3? panels should be reorganized.
221
Where are presented this cell type specific data?
227
"The mucus layer is reduced 100 % from 8 μm to 0 μm (Fig. 3 E-F)" figure panels should be reorganized to be presented sequentially.
240
The increase of ER stress in ben/ben mpi mice was presented before.
244
in which mice strain did you observe apoptosis?
250
Where are these WGA stainings presented? Figure 4A should be indicated.
256
Figure 3G panel should be moved to follow the description in results
267
The magnification provided do not allow to visualize the loss of mitochondria.
Where are presented the data related to the size of microvilli? Same question for the small intestine observations.
271
Was apoptosis also increased in the colon goblet cells?
276
where are the ben/ben mice apoptosis data presented?
278
the verb is lacking
281
The IL10 decrease is not significant
284
The reference to the figure is not correct, there is a confusion with the other experiments in ben/ben mice...
304
The microvilli observations are still not presented here for the small intestine. Same comment for goblet cells. All these data should be presented.
312
Table 2 is not provided
314

The changes in gene expression are not significant according to what is presented in the figure
324
It is not clear whether proteomics was performed only in the small intestine or also in the colon.
325
where are these data presented?
327
The changes in gene expression are not significant according to what is presented in the figure
340
Is proteomics results (7B) performed in the colon as written in line 340 or in the ileum as written in the figure legend?
344
Experiments with tunicamycin in HT29 MTX cells are not presented in the methods section.
What type of glycosylation is inhibited by tunicamycin? This information should be added.
358
There are no data provided showing that the loss of Mpi was worse than loss of O glycosylation and the comparison with the phenotype of MUC2 KO mice was not evaluated experimentally either.
370
The death of mice 2-3 weeks after deletion of Mpi was not presented in the results.
372
References should be provided for this mice collection
383
Reference for this work should be provided
392-399
References should be provided
450
where was this effect of mannose supplementation reported? There is no reference.
451-460
Here again no references are provided.
482
Supplementary file was not provided

Figure 2G: goblet cells were counted in the colon crypts, not villi.

Figure 4E: statistical analyses are not shown

Figure 7D: groups are interchanged

Table 1: all working dilutions of antibodies should be provided for reproducibility.

Table 3: LS1745 and HT29-MTX were not produced for this paper.

Reviewer #3

(Remarks to the Author)
Major comments concerning the proteomics part only:
• In your proteomics methods part, you state that you prepared the proteomics samples as previously described by Birchenough et al., yet the publication you refer to did not perform proteomics from gel bands, nor from intestinal tissue, but from mucus, which intrinsically calls for a different sample preparation. You state that gel bands were sent in for proteomics measurement, yet it is unclear why the samples were run on a gel at all, and it is also not clear which bands were excised.
• You further fail to provide important information concerning the proteomics experiment, like how many biological replicates were used, how the samples were lysed, if and how they were reduced, alkylated, or digested. You provide no information on the instrumentation (the LC system used, the solvents, chromatography column, gradient, the ion source, the mass spectrometer, or the acquisition mode). You also provide no information about the data analysis of the resulting proteomics data, like which software was used, which database you searched against, your FDR control, your mode of quantitation, your used statistics, etc.
• Moreover, the proteomics data should be made available for the reviewers through submission to a proteomics repository (e.g., the PRoteomics IDEntifications (PRIDE) Database by the ProteomeXchange Consortium): https://www.proteomexchange.org/ (like they did in the publication you refer to in your proteomics method section) – this is good practice in the proteomics community.
• In the results section discussing the proteomics data, you refer to Table 2 (line 312), which is not provided. The plot shown in Figure 7B is not a common way to visualize proteomics/peptidomics data. The names refer to proteins, did you analyze proteins or peptides? Moreover, it is not explained anywhere what the green triangles or red dots in Figure 7B represent. How many proteins/peptides were identified in total? How many of them are differentially regulated, to which extent and significance level (multitesting corrected)? How many biological replicates were used?
• In lines 336 – 340, you state that you performed proteomics analysis on colon lysates, and refer to Figure 7B, which,

according to the legend, is peptidomics of the small soluble fraction of the terminal ileum. Please elaborate which statement is correct.

Taken together, there is insufficient information about the proteomics experiment to review it properly on a technical level. Furthermore, it is unclear what exactly was analyzed and is shown in Figure 7B, and no additional information (i.e. protein tables) is provided. The proteomics part of this publication does not meet the standard required for publication.

General comment: Overall the manuscript is hard to read, especially the introduction contains only fragments of sentences and lacks references. Mouse models and abbreviations are not explained upon first mentioning them. The method section (in part, e.g., proteomics/peptidomics, mouse models, primers, antibodies (ATF4 for IHC), table 2 and supplements are lacking.

Reviewer #4

(Remarks to the Author)
I co-reviewed this manuscript with one of the reviewers who provided the listed reports. This is part of the Nature Communications initiative to facilitate training in peer review and to provide appropriate recognition for Early Career Researchers who co-review manuscripts.

Version 1:

Reviewer comments:

Reviewer #1

(Remarks to the Author)
The authors have addressed our previous comments thoughtfully. Most of our original concerns related to the clarity of writing and interpretation rather than the experimental results themselves, and the revised manuscript reflects meaningful improvements in these areas. The edits indicate that the authors understood our feedback and implemented it effectively.

Reviewer #2

(Remarks to the Author)
Although the authors have addressed most of my previous comments, the quality of the manuscript remains below the standards expected for publication in Nature Communications, particularly with regard to the writing of the introduction, the details of the materials and methods to ensure reproducibility, and the overall care taken in the writing. This manuscript has not been carefully proofread by the authors.

The overall study design is clearer now that another article from the authors was published in JCI Insights (https://doi.org/10.1172/jci.insight.180752) to present the mouse MPI KO models. However, I feel that the submission of the previous version of the current study was pre-matured, since it represented an unnecessary hard reviewing process due to undisclosed information.

The small intestine and colonic phenotype of the MPI hypomorphic and intestinal conditional KO mice was already described in part in the JCI insight article (presence of abnormal goblet cells and lower mucus production). The current study expands these observations, including in an inflammatory context by using DSS model and by searching underlying cellular/molecular changes.

Specific comments:

1. The viability data of the conditional KO should be presented in the methods or results but not in the introduction.
2. Figure 1D: villus should be replaced by crypts (also in figure 2F). Line 806: Replace “CRIPSR Cas 9 targeted mice” by a more specific designation or remove. “Het = heterozygote, Homo = homozygote » are not used in the figure, to be removed.
3. Figure 3: the legend is missing in 3D (MPI F/F or villin). Line 851: remove “repeated five times”?
4. Figure 4F and G: statistical analyses are missing for mitochondrial activity data.
5. Figure 5: all abbreviations should be defined in the legend. Figure 5B: the tissue where these genes were measured should be indicated.
6. Figure 6: Line 900: can you confirm this is colon and not small intestine as indicated? 904: should villus tip be corrected to crypt?
7. Data availability: the database where the data were deposited should be indicated (SRA for microbiome?). Idem for proteomics data.
8. Title: I think that the title should use more specific wording than “host microbe equilibrium” and “gut immune balance”. Metabolism is also vague, it should be specified epithelial cell metabolism.
9. The introduction still lacks clarity. For instance, Lines 108 - 119, it is not clear whether the authors refer to their previous or current publication. Line 47: this sentence suggests that there was a global MPI KO model while it was hypomorphic only at the whole-body level. Line 83: congenital disorders of glycosylation are defined twice. Line 87: there is no verb. Line 94 : the role of MPI was already defined line 87. The introduction should be reorganized since it is still hard to read.

10. In the material and methods, all suppliers and catalogue number should be provided to ensure reproducibility. Most of them are still missing in the current version.
11. Line 182: Did the DSS treatment lasted 5 or 7 days?
12. The methods for the culture of cell lines are not sufficiently described (lines 189-193). There is no detailed information related to the generation of KO cell lines. All references to commercial products should be provided. “and LS174T-Mpi KO cell lines 10mg/ml puromycin 191 was added” should be corrected.
13. Add MPI ben mice in the microbiome method section (line 195)
14. L245: the sentence should be corrected: “fluorescently labeled 16s RNA probes conjugated with in Hybridization buffer”. That type of typo are still frequent in the manuscript.
15. Line 263: indicate which cells were used in the searhorse assay.
16. Line 298: the reference for the scRNAseq data is not appropriate (Uhlen, M. et al. A genome-wide transcriptomic analysis of protein-coding genes in 726 human blood cells. Science (1979) 366, (2019).) The methods used to analyze these scRNAseq data are missing.
17. Line 315: a space is missing after baseline
18. Line 301: replace Intestinal by colon to match with the figure 1C and D.
19. As indicated in my previous review, this sentence is misleading “To test whether the colitis phenotype was intrinsic to the colon” (Line 332) because MPI is deleted in all intestinal epithelial cells, not only in the colon.
20. Figure 2K: the meaning of 136-7 and 1049-8 should be explained in the legend.
21. Line 341 “terminal” should be corrected
22. Figure 4E: the legend is missing (color code in the figure).
23. Line 399-408: these data related to the spontaneous colitis should be presented together with figure 2 to keep a logical flow.
24. Stars indicating significance were added in the figure 5B for IL10 when compared to the previous version while you added in the text that this decrease was not significant (Line 408). This is highly confusing on how statistical analyses were performed.
25. Line 422-426: the results of the microbiota of MPI ben mice is discussed here, the divergence between the two models (CKO or ben) should be discussed since it would be expected that the intestinal MPI drives most of the effects.
26. Line 429: there is no verb in the title
27. There is no capital letter in “goblet cell” (to be changed in all the manuscript)
28. Line 440: “detect any change in the abundance of the small soluble molecules”, I guess you mean protein or peptides?
29. Line 443: why do you only comment the decrease in Reg3g while other AMP are also reduced in qPCR analysis?
30. Revise: “compared to wild type. Including –SDH and Ndufa10 all decreased by 10-20- fold (Fig. 7G).” Line 461
31. Line 467-473: In this paragraph it is highly confusing whether these data were obtained in the colon or ileum. You are referring to the same figure for the colon as the one you referred before for the ileum (Figure 7B).
32. 478: the figure panel 4F should be moved to in figure 7 to follow the description of the results.
33. 528 “bacteria break down mucin O-glycans or block quarum sensing” I guess you mean quorum sensing but I do not understand the link with the discussion in this context.
34. 565 : Correct : “oxidative phosphorylation 27 ,28 , 29). Glycosylation could impact mitochondrial proteins due to changes in protein folding and stability 30)”
35. 571: “Paneth cells secrete anti-microbial peptides stored in dense granules at the base of small 571 intestine villi” In this case, crypt should not be corrected to villi.
36. Line 581: Revise: Gfi1 (Stappenbech)
37. Line 923: Do you mean markers of Paneth cells instead of goblet cells?
38. Line 959: (Top)- there is no bottom panel?

Reviewer #3

(Remarks to the Author)
Remaining points for the proteomics part only:
1) The information that you just performed a clean-up per gel is still missing in the manuscript. Now it reads as if you would have run a whole SDS-PAGE separation.
2) Your number of replicates is very low with 3 mice in a group. In addition, your method description still lacks the information how you did your quantitative analysis and statistics to determine significant protein changes.
3) The information of how to access your proteomic data is missing in the manuscript. While you report the accession number, there is no information about where you uploaded the data, and with the accession number alone, there is no way to access the data.
4) The visualization in Fig. 7B lacks in providing information about statistical significance of observed changes (in comparison to e.g., a volcano plot). Moreover, the description of the axes appears faulty (brackets wrong in y, why is the intensity in x (which intensity is meant? Or are these spectral counts?) reported as exponential with a base of 2?). The now added Table 2 only lists a few selected targets and it is unclear why these were selected to be reported and whether their abundance change is significant. In the source files one can find the whole protein table which should make it into the supplement after addition of statistical analysis results which are also missing in the current table.

Reviewer #4

(Remarks to the Author)
I co-reviewed this manuscript with one of the reviewers who provided the listed reports. This is part of the Nature Communications initiative to facilitate training in peer review and to provide appropriate recognition for Early Career Researchers who co-review manuscripts.

Version 2:

Reviewer comments:

Reviewer #2

(Remarks to the Author)
1. As explained in their letter, the authors have re-analysed the qPCR data and identified calculation errors in several figure panels (3B, 4D, 5E, 7C, D and G). In several cases, statistical significance has been lost compared to the previous version. In addition to the changes in qPCR calculations mentioned by the authors, I noticed that the number of samples/dots per group (n = 5) in the new Figure 3 (formerly Figure 5) is different to that in the previous version (n = 7). Furthermore, the legend of Figure 3 still states that n = 7 (line 1029). The authors should explain why data were excluded between the two versions.
2. Similarly, in Figure 7C, there are only 5 dots in the graphs but n=7 is indicated in the figure legend (line 1122). The number of replicates also changed compared to the previous version in Figure 7G, for instance for FASN there was 7 replicates for the control and 5 replicates for the KO, while in the new version there are 6 replicates in each group. Does this mean that samples were mixed between groups in previous version?
3. In the new version, the y axis of some qPCR data is ‘Fold change compared to Gapdh’, but this wording should be corrected to ‘mRNA level’ or ‘mRNA expression’ since the expression level is normalized to Gapdh but there is no fold change calculation.
4. Line 330: “This strain was previously described” a reference should be provided.
5. Line 335: correct “havespontaneous colitis”
6. Line 371: ‘The culmination of these cellular stressors is goblet cell death in the ileum’ and line 378 ‘the cumulative effect of Mpi deficiency is higher ER stress and some goblet cell apoptosis in the ileum’, the structure of this repetitive paragraph should be improved.
7. Figure 5F and G: The representation of statistical results does not show which groups are significantly different.
8. Figure 6A: the lower panel is hidden by the Figure 6B.
9. Line 428: ‘The Mpi ΔVillin phenotype had larger microbial shifts than the Mpi ben microbiome but the compositional changes were similar’, this conclusion is not valid since there is no significant differences between the control and Mpi ben mice. Line 430: “indicates they are more sensitive to mucus regulation» this statement is over speculative and should be removed.
10. Line 449: “terminal ilium » to be corrected (idem line 450).

For several of my comments, the authors state having made the modifications, but it appears not to be the case in the manuscript:
11. In figure 1D and 2F: villus should be replaced by crypts since there is no villi in the colon. I have already requested this change in the previous version but the authors did not apply it.
12. Line 313: the reference for the single cell atlas has not been changed. The is a PMID (28818976) written in the text and it is not a relevant publication.

Reviewer #3

(Remarks to the Author)
Thank you for cleaning up the proteomics part. The experimental design of the proteomics part is rather poor with n=1 and two years later n=2, with the main proteomics figure (7B) being based on a single experiment. Your two statements in your rebuttal that you did not perform statistical testing on the proteomics data and that you performed a screen of potentially altered proteins that were significantly altered (<10% mutant) and fit a similar pathway are contradictory. If you did not perform statistical testing you cannot determine significantly altered proteins. The method for quantitation is not clear. Did you use ion intensity (Fig. 7B) or spectral counting (Suppl. Fig. 3A) for quantification?

I would like to add a few additional comments to the lipidomics part since you have drawn my attention to it:
1) I would not call this lipidomics or even a lipidomics screen as you did in the main manuscript (line 488), but total fatty acid profiling, since you hydrolyzed all complex lipids and analyzed just the total fatty acids, as described in the supplement, lines 1201-1236.
2) Please clarify whether the fatty acid profile you report in supplemental figure 3 is generated from ileal tissue (line 488) or

serum (line 1206).
3) Fig 3 C: What does the nd above the bars mean? Do you mean ns, non-significant? Please correct.
4) line 474: Please correct Acyl-CoA to Acetyl-CoA and delete triglycerides. You only show fatty acid de novo synthesis not triglyceride synthesis proteins.

Reviewer #4

(Remarks to the Author)
I co-reviewed this manuscript with one of the reviewers who provided the listed reports. This is part of the Nature Communications initiative to facilitate training in peer review and to provide appropriate recognition for Early Career Researchers who co-review manuscripts.

Version 3:

Reviewer comments:

Reviewer #2

(Remarks to the Author)
The authors adequately adressed my last comments.

Reviewer #3

(Remarks to the Author)
Thank you for addressing all my points of criticism.

Open Access This Peer Review File is licensed under a Creative Commons Attribution 4.0 International License, which permits use, sharing, adaptation, distribution and reproduction in any medium or format, as long as you give appropriate credit to the original author(s) and the source, provide a link to the Creative Commons license, and indicate if changes were made.
In cases where reviewers are anonymous, credit should be given to 'Anonymous Referee' and the source.
The images or other third party material in this Peer Review File are included in the article's Creative Commons license, unless indicated otherwise in a credit line to the material. If material is not included in the article's Creative Commons license and your intended use is not permitted by statutory regulation or exceeds the permitted use, you will need to obtain permission directly from the copyright holder.
To view a copy of this license, visit https://creativecommons.org/licenses/by/4.0/

REVIEWER RESPONSE

The authors would first of all like to thank the reviewers for all of their time for critical and helpful comments to improve the quality of the manuscript. We were trying to walk the fine line of providing enough information about the original strain without self-plagiarizing, so important information thus appears to have been left out. The paper describing the original ENU-mutant strain (*benadryl)* will be published in mid-July at JCI Insight, which should improve the context. However, we also acknowledge significant readability issues were found and we have made a significant re-write to improve clarity and readability.

We have provided a point-by-point response below with author responses in red text.

Changes in the text are highlighted for Reviewer 1 and 4, Reviewer 2, and Reviewer 3.

Reviewer #1 (Remarks to the Author):

Key results
The manuscript by Roy and SoRelle et al. establishes that dampened mannose production (a surrogate for N-glycosylation), as observed in congenital disorders of glycosylation, can compromise intestinal homeostasis to the same extent as MUC2 knockout, a finding which elevates the importance of mucin N-glycosylation beyond the understood functions of ER transport and dimerization. The authors achieve this through a first-in-class murine model of mannose phosphate isomerase deficiency, with both general and intestine specific knockout strains being employed to confirm goblet cell reduction, severe colon pathology, elevated ER stress, limited mucin production, increased inflammation, and dysbiosis as a direct outcome of limited N-glycosylation. The murine strains presented thus represent both a step forward in understanding the role of N-glycosylation in production of protective MUC2 and antimicrobial peptides, and in providing a robust system by which to screen therapeutic options.

Taken together, this work represents a significant advance for the field, and we recommend publication provided that the following points are addressed.

We thank the reviewers for their complementary comments and feedback on our report. All the corrections have been incorporated in the manuscript, and changes have been highlighted in green.

Validity:
The data provided in figures 3B and 3D show elevated expression of specific ER stress response genes in their MPI knockout mice; however, given the established importance of N-glycosylation for general ER proteostasis and chaperone activity (1), can the authors confidently claim that this is due specifically to Muc2 accumulation/misfolding?
"Cryo EM and glycopeptide mass spectrometry studies have shown Muc2 C terminus N-glycans influence the shape of the protein as these glycans line the dimerization domain21.

Improper glycoprotein folding can prevent ER-Golgi progression. Consequently, it appears Muc2 accumulates in the endoplasmic reticulum increasing ER stress markers of AT4, BiP, CHOP and Grp94 by mRNA expression in benadryl mice after DSS challenge (Fig 3B) and in MpiΔVillin at baseline (Fig 3D). "

We agree that multiple glycoproteins could be retained in the ER and lead to ER stress. While Muc2 is the predominant one being secreted, our data do not definitely show it is the only one. We have reworded in lines 337-342:

"Cryo EM and glycopeptide mass spectrometry studies have shown Muc2 C terminal N-glycans influence the shape of the protein as these glycans line the dimerization domain[32]. Improper glycoprotein folding can prevent ER-Golgi progression, and while Muc2 is the an abundant intestinal glycoprotein synthesized, other glycoproteins could experience similar ER retention. Therefore, we measured mRNA expression of ER stress markers and found increased AT4, BiP, CHOP and Grp94 in benadrl mice after DSS challenge (Fig 3B) and in $Mpi^{\Delta Villin}$ mice at baseline (Fig 3A,B). "

Significance:
We recommend the authors better connect the following four statements of significance above with their results, which were focused on a model of induced colitis starting from a point of CDG-MPI Mannose deficiency. We furthermore do not see how the data presented could establish N-glycosylation as more significant than O-glycosylation for "maintaining intestinal homeostasis." The manuscript establishes mannose production (a surrogate for N-glycosylation) as essential to the production of mucin, a finding of great significance, but it is established in the literature that mucin attributes much of its microbe feeding and modulating activity, when formed, to the presence and distribution of specific O-glycan structures (2-6).

The reviewer's point is well taken, and we have re-worded our statements to indicate that it appears N-glycosylation is a prerequisite for O-glycosylation (line 486). In part, because N-glycosylation is essential for Muc2 to migrate to the Golgi where O-glycosylation occurs.

1. "While O-glycosylation makes up >80% of the weight of the glycoprotein, N-glycosylation has been shown to be important for subsequent dimerization and maturation in the Golgi.

Reference added to cell line studies showing N-glycosylation inhibition by tunicamycin retains Muc2 in the ER, which prevents it from being glycosylated in the Golgi (line 332). We also added a sentence linking Mpi deficiency with lower N-glycosyaltion with a reference to our upcoming paper. We would attempt the experiment of EndoH digestion on Muc2 from the colons of $Mpi^{\Delta Villin}$ mice (to prove ER retention), but Muc2 levels are undetectable by Western blot.

Changed to (line 330-334): "While O-glycosylation makes up >80% of the weight of the glycoprotein, N-glycosylation has been shown to be important for subsequent dimerization

and maturation in the Golgi.[30] Our previous study found that an ENU-induced allele affecting Mpi enzymatic activity increased unoccupied N-glycan sites by 554%.[30,31]"

2. "Furthermore, mucins provide nutrition to microbes to keep them in a well- balanced equilibrium with the host. Comprising 80% of the weight of mucins, carbohydrate glycans are the nutritional source for commensal bacteria. Loss of Muc2 O-glycans secondary to N-glycan loss creates concern for dysbiosis. Dysbiosis, an imbalance in the gut microbiome, can drive disease by bacteria penetrating the epithelial barrier or secretion of altered metabolites. This work addresses long standing questions concerning N-glycan function for mucins in vivo. As mucins are 80% O-glycans by weight, defects affecting these post-translational modifications were thought to be more impactful. However, we have discovered that N- glycans are even more important in Muc2 maturation as genetic deficiency of Mpi causing N-glycan loss leads to a phenotype as strong as Muc2 deletion."

Line 402-406: Furthermore, mucins provide nutrition to microbes to keep them in a well-balanced equilibrium with the host.[33] Comprising 80% of the weight of mucins, carbohydrate glycans are the nutritional source for commensal bacteria. Dysbiosis, an imbalance in the gut microbiome, can drive disease by bacteria penetrating the epithelial barrier or secretion of altered metabolites.

Lines 477-487: This work addresses long standing questions concerning N-glycan function for mucins in vivo. As mucins are 80% O-glycans by weight, defects affecting these post-translational modifications were a primary focus of research. However, we have discovered that N- glycans are a prerequisite for Muc2 maturation as genetic deficiency of Mpi causing N-glycan loss prevents any O-glycosylation. Using Alcian blue staining as a surrogate for Muc2 maturation, mutations affecting core 1- or core 3- O-glycans ($C1galt1^{f/f}$; $Villin^{Cre}$, $C3Gnt^{-/-}$) or Golgi transport (*Trvp23b*, *Yipf3*) still retain some Alcian blue staining. However, when Mpi is deleted, there is no Alcian blue staining left, which resembles a $Muc2^{-/-}$ strain or complete loss of O-glycans (double knockout of $C1galt1^{f/f}$; $Villin^{Cre}$ and $C3Gnt^{-/-}$).[21,27,26]

3. "While immunohistochemistry can detect residual Muc2 in MpiΔVillin mice, remarkable lack of mucus barrier thickness, concomitant bacterial invasion, and dysbiosis. We found the residual Muc2 was no O-glycosylated, which is a mechanism for its gel-like properties that underly barrier functionality. Since >90% of glycans on Muc2 are O-glycans, the role of N-glycans has been understudied. "

This modification clears up language and ties in the changes to a loss in Mpi more directly than the extrapolation to N-glycans themselves.

Lines 513-519: Although we could detect residual Muc2 in the $Mpi^{\Delta Villin}$ colons by IHC, no O-glycans were detectable by DBA-lectin staining nor N-glycans in epithelial cells by WGA-lectin staining. Therefore, we found low levels of Mpi attenuates O-glycosylation, but

deletion of Mpi prevents any O-glycosylation formation in the intestinal epithelial cells. Muc2 is attenuated in the Mpi hypomorphic allele, and is nearly absent by IHC in the $Mpi^{\Delta Villin}$ mice.

4. "several congenital disorders of glycosylation (including MPI-CDG) and now has been linked to loss of mucin protection. Therefore, while mucins are heavily O-glycosylated, N-glycans have an essential role in maintaining intestinal homeostasis."

This modification ties in that N-glycans are not more important than O-glycans, just simply an earlier step that needs to be accomplished in maturation.

Lines 623-626: Several congenital disorders of glycosylation (including MPI-CDG) have been linked to decreased mucus production, but this study provides evidence that N-glycosylation is a necessary precondition before important O-glycans can be added.

Data and methodology:
The quality and presentation of the data are sound.
Thank you

Analytical approach:
We find the analysis of the presented data to be sound.
Thank you

Clarity and context:
Two following statements in the results and discussion should be referenced if the prior work of the authors is used to make the argument.
1. "During an ENU-mutagenesis forward genetic screen, a mouse strain named benadryl associated with a visible and allergic phenotype (Fig. 1 A,B, p=1.6x10-12, recessive). This strain mapped to a missense variant p.H54R in the mannose phosphate isomerase gene named Mpi. We have previously described this strain, which is notable for recreating most features of the human congenital disorder of glycosylation MPI-CDG including congenital diarrhea…"
2. "We use the term N-glycan KO 397 because our previous work showed Mpi deficient mice had N- glycan sites without any carbohydrate at glycan sites (by glycopeptide analysis)."

Now referenced with the July 22nd article coming from JCI Insight.

The Introduction is difficult to understand and has poor syntax (see the specific examples below). The results and discussion sections present stronger arguments, but it is hard to

defend the impact of this work without a well-written introduction to provide the necessary context.
Examples:
1. "The role of the mucins in the gut is not only restricted for their protective function but also impact the dynamics of the changing microbiome owing to the mucus-bacterial symbiotic relationship."
2. "One of the prominent O-glycosylated family of mucins lining gut epithelium is MUC2 and glycosylation status determines the gel-forming property of MUC2 in the form of mucus is essential for the gut protective barrier"

We thank the reviewers for their significant effort to improve the readability of the manuscript. We have made substantial re-writes that should clarify our points and improve our scientific communication.

Line 62-64: Mucins in the gut not only serve a protective role but also influence the evolving microbiome through their symbiotic relationship with bacteria in the mucus layer.

Line 68-70: MUC2 is a prominent member of the O-glycosylated mucin family that lines the gut epithelium, and its glycosylation status is crucial for its gel-forming properties, which are essential for maintaining the protective mucus barrier.

References
We recommend adding further references in the specific areas above. Below are the references that we are citing in this review:
1. Nat. Chem. Biol. 10, 902–910 (2014). https://doi.org/10.1038/nchembio.1651
2. Nat. Microbiol. 6, 574–583 (2021). https://doi.org/10.1038/s41564-021-00876-1
3. Nat Chem Biol 19, 778–789 (2023). https://doi.org/10.1038/s41589-023-01272-y
4. Cell Host Microbe 31, 1371-1385 (2023). https://doi.org/10.1016/j.chom.2023.07.003
5. Sci Rep 13, 13982 (2023). https://doi.org/10.1038/s41598-023-40497-8
6. Bergstrom, K., & Xia, L. (2022). Gut Microbes, 14. https://doi.org/10.1080/19490976.2022.2052699

We thank the reviewers for their helpful citations of recent literature. We are more specialized in areas of glycobiology and immunology and want to be sure appropriate studies are cited and given credit. They have now been added as references: 4, 28, 33, 34, 36, 40

Reviewer #2 (Remarks to the Author)

The manuscript by Roy et al evaluated the role of the N-glycosylation enzyme Mpi in intestinal homeostasis. This work is relevant to congenital disorders caused by Mpi mutations and to understanding the role of N-glycosylation for gut health. The experiments are based on i) Mpi hypomorphic mice obtained by mutagenesis or ii) specific deletion (KO)

of Mpi in intestinal epithelial cells. The results show that Mpi is crucial for mucus secretion and homeostasis of goblet and Paneth cells. Disruption of Mpi leads to dysbiosis and inflammation. These data are novel and expand the understanding of the importance of N-glycoslyation in the intestine and may lead to novel therapeutic strategies. The results seemed to be technically sound and state of the art methods were used.
We thank the reviewers for their complementary comments and feedback on our report.

However, the overall quality of the manuscript is suboptimal since there are many typos, some sections appear to have been copied and pasted from other documents, the order of the figure panels does not follow the description of the results, some experiments described in the materials and methods are not presented in the results (and vice versa) and the discussion critically lacks references to previous work. Moreover, the procedure used to generate the mouse models is not detailed in sufficient detail. All these important limitations of the manuscript can be easily corrected after a careful proofreading.

Response: We thank the reviewers (especially R2) for their significant effort to improve the readability of the manuscript. We have made substantial re-writes and proofreading that should clarify our points and improve our scientific communication. All the corrections have been incorporated in the manuscript, and changes have been highlighted in yellow.

1. Previous studies using Mpi total KO mice (e.g. 10.1074/jbc.M511982200) are not mentioned in the manuscript and the lethal phenotype of the total KO should be presented to justify the choice to focus on a hypomorphic model. Knowing the phenotype of the total KO mice, the authors should provide the litter size and/or mortality rates in their hypomorphic and conditional KO mice.

Response:This is a good point, and we have added a description of the Mpi-KO allele to explain why we focused on the hypomorph allele.

Lines 97-106: In a forward genetic screen in mice, an *N*-ethyl-*N*-nitrosourea (ENU)-induced point mutation in *Mpi* (p.H54R) was found that maps to an allergic phenotype named *benadrl.*[16–18] This allele was reproduced by CRISPR knock-in which was denoted in the study as hypomorphic $Mpi^{benadrl\ (ben)}$ (Mpi knockout mice are embryonic lethal).[18] Enzyme activity assays determined Mpi protein was unchanged but enzyme activity was only 5% in homozygous mice due to a 20x weaker substrate binding affinity. Mutations in *Mpi* alter glycosylation affecting most physiological systems; we found in this study that downregulation of MPI leads to phenotypes that are attributable to intestinal epithelial hypo-N-glycosylation. One of the principal symptoms in human patients is diarrhea; here, we found loss of goblet cells in the large intestines of benadrl mice, which could lead to intestinal malabsorption or PLE.[11,18,19]

The birth rate and mortality of the Mpi-benadryl strain is present in our JCI insight publication. This data has now been added for the CKO strain:
Lines 110-113: $Mpi^{\Delta Villin}$ mice were born at 50% of the expected Mendelian ratio of 25% ($Mpi^{f/f}$ x $Mpi^{f/+}$; $Villin^{Cre}$) and were not fertile. In the barrier facility, $Mpi^{\Delta Villin}$ mice survived well through 4 months, but in conventional housing, most died in 6-8 weeks."

Figure 1B: This panel was replaced as it was found to have duplicative data with panel 2C. The original G3 mouse screening data is shown now. This has been corrected on the raw data submission as well.

2. The authors use the term "Benadryl strain" or "Benadryl allele" in the introduction and materials and methods to refer to the Mpi hypomorphic model. I understand that this is the designation chosen by the authors to name the phenotype of these mice but this is not clearly explained and this name may be misleading given the antihistamine effect of this drug, which is unrelated to the current study.

Yes, this is challenging. We tried to walk the line between avoiding self-plagerism and providing enough detail. This has been addressed above in Comment 1.

3. More experimental details should be provided regarding the generation of the transgenic mice. The choice of the candidate Mpi105 mutation (p.H54R) should be justified in the methods. I actually understood much better how this mutation was identified when I read the introduction of another publication by the authors (10.1038/s41467-023-39398) where their forward genetic screen is much more clearly explained. What are the functional consequences of the studied SNP mutation on Mpi activity? How did the authors exclude mutations other than in Mpi in the ben/ben mice?
This has been addressed above in Comment 1.

4. The authors state that the mutation was knocked-in (Line 106) which is confusing since mutations were generated with ENU mutagenesis. The authors refer to an unpublished procedure (Lin et al, submitted supplement). There are also mention of "CRISPR Cas9 targeted mice", for instance in the legend of Figure 1. All these procedures should be clarified.
This has been addressed above in Comment 1.

5. It is stated at the end of the introduction that "benadryl strain displayed reversal of intestinal physiological disorders simply by administration of Mannose" but no experimental data support this claim. This experiment with mannose supplementation would have indeed clearly reinforced the significance of the study by providing therapeutic perspectives but these data seems to have been removed from the manuscript or maybe presented elsewhere but without providing the reference. The mannose content of the diet fed to the mice should be reported since it may highly influence the phenotype of Mpi deficiency.

This mannose response data will be published in the JCI Insight paper on July 22nd. We had added a reference to this study now. The mannose rescue mechanism will be further explored in a future study.

This is a good point that dietary mannose could influence the phenotype. There is no mannose present in the standard mouse chow diet (as it is found in fruits and berries predominately, and confirmed on dietary chow nutrition information), so dietary mannose is unlikely to ameliorate the phenotype. We have added this data to the manuscript on line 597-598.

6. In the tissue specific mouse model of Mpi deletion, the authors use the term "intestine-specific N-glycan deficiency"/"intestinal specific KO", whereas the deletion of Mpi was performed specifically in the intestinal epithelium (Villin Cre mice). The manuscript should be corrected to clearly state that the knockout was restricted to the intestinal epithelium, while N-glycosylation remains functional in other intestinal cells. The authors state "to test whether the colitis phenotype was intrinsic to the colon, we used an intestinal epithelial cell specific knockout (Mpi flox/flox ; VillinCre : MpiΔVillin)" (Line 223). However, Villin is also expressed in the small intestinal epithelium and the phenotype cannot be attributed to the loss of Mpi in the colon only. There is also a confusion in the manuscript between villi (small intestine) and crypts (colon and small intestine), see for instance line 300 "colonic villi".

We thank the reviewer for helping us correct our language to be most accurate. The following changes have been made:

Line 45, 107: "intestinal epithelial-specific N-glycan deficiency"

Line 316-319: "to test whether the colitis phenotype was intrinsic to the intestinal epithelium, we used an intestinal epithelial cell specific knockout ($Mpi^{flox/flox}$; $Villin^{Cre}$: $Mpi^{\Delta Villin}$)"

line 422, Fig 1D, 2G, 3F legend: Villi changed to "crypts".

Line 428, 566, Fig 6B legend: Small intestine "crypts" changed to villi

Line 380-381: villi changed to "microvilli"

7. There is no information provided in this manuscript regarding the type of intestinal epithelial cells that express Mpi. This information could be retrieved from scRNA-seq dataset and would help to understand the cellular mechanisms of the KO.
We retrieved single cell RNA-seq data sets from the Human Protein Atlas, which shows MPI expression is enhanced in the proximal enterocyte cell type. It is also present at significant levels in Paneth cells, Goblet cells, and distal enterocytes. This high expression in these tissues may indicate why they are so strongly affected. Each of the cell types were affected by the loss of Mpi in the mouse model. We have added a section describing this in the first results section to provide a rationale for our studies.

Line 293-296: To understand the cellular mechanisms of Mpi loss in the intestine, we compared *MPI* expression in single cell RNA seq data sets of human tissues. *MPI* RNA was

highest in proximal enterocytes, but goblet cells, Paneth cells and distal enterocytes each expressed significant levels of MPI RNA.[25]

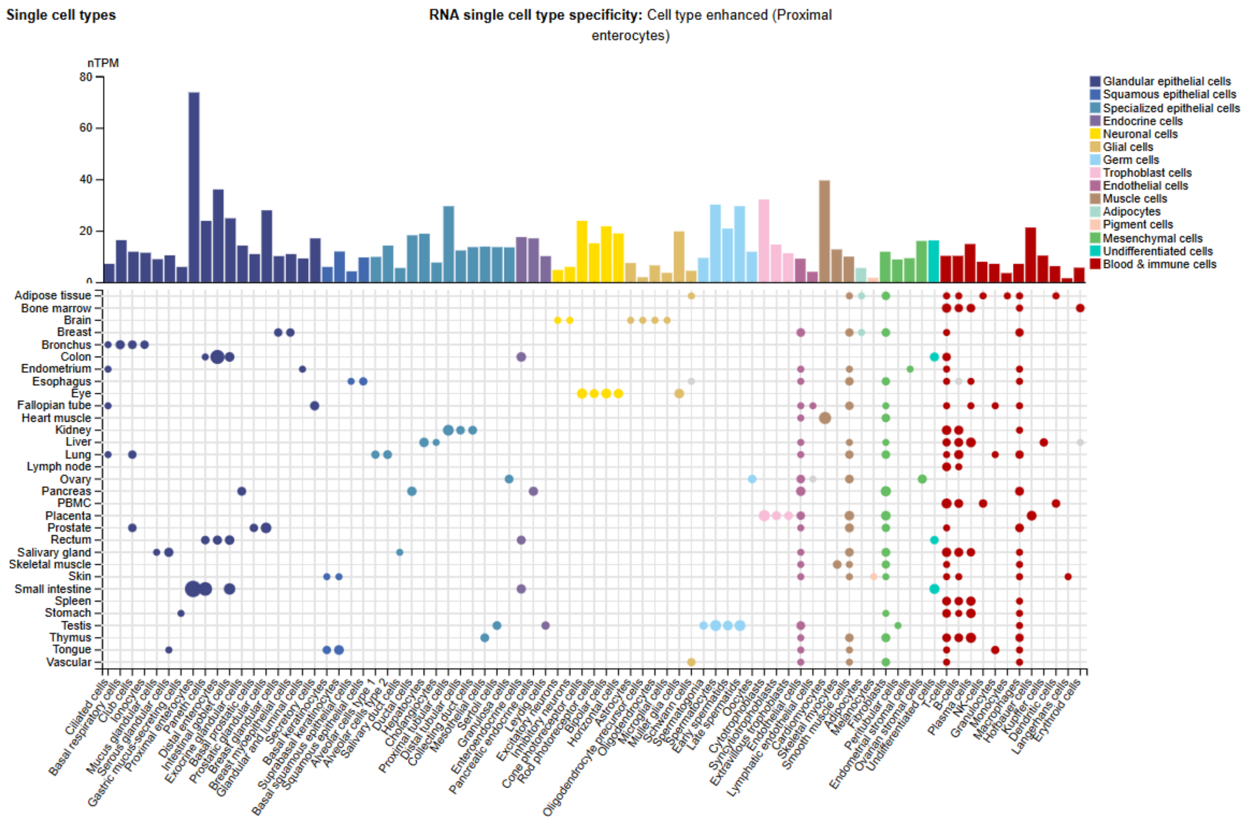

8. The gut microbiota composition was presented in the intestinal epithelium Mpi KO mice but not in ben/ben mice. These data should be provided to link Mpi activity with microbial changes.

Response: The gut microbiota composition for the Mpi$^{ben/ben}$ mice have been included in Supplementary Fig 2 (see below).

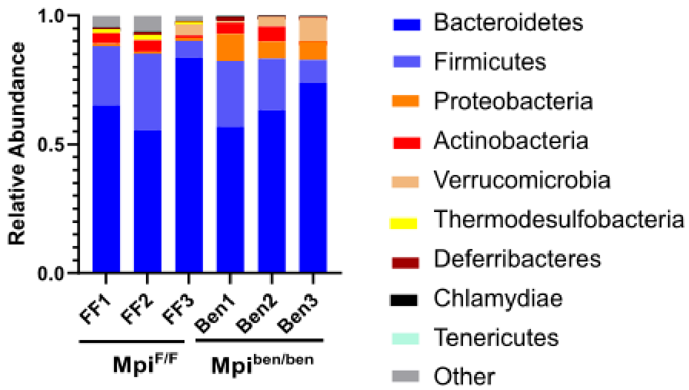

10. **The format of the reference is not the same in the introduction and materials and methods.**
The materials and methods lack critical details (indicated below) and contains many typos and should be carefully corrected.

Response: Reference formatting was aligned with Nature journal standards and additional experimental information has been incorporated in the material and methods section.

11. **One example of the lack of care taken in drafting the manuscript:**
In legend of Figure 6 " Microbiome sequencing of Mpi F/F and MpiΔVillin (Update the labels to be consistent- you can mark them as 1, 2, 3 but put Mpi F/F below with a line to indicate they are in the same group".

Response: Thank you, labeling has been updated to be more clear in Figure 6A, and statistical analysis was added to the figure legend.

12. There is no statistical analysis section. The number and age of mice used in each experiment is not provided in the methods section.

Response: Data Reproducibility and Statistical analysis section have been included in manuscript. Also, the number and age of mice used in each experiment have been provided in methods section.

Lines 268-273

13. **There is no data availability section (e.g. microbiome, proteomics) and supplementary materials are not provided.**
Response: Data availability section, supplementary data, and raw data added: Lines 275-279

14. **The figure panels are not presented in the order of the description in the results which makes the reading much more difficult.**

Response: The figure panels have been rearranged to make it easy for the reader to interpret the results.

15. Some experiments related to LS174T cell line MPI KO are presented in the materials and methods section and in figure S1 but are not described in the results.

The LS174T cell line data are now presented in Figure 2 to complement the panels describing loss of Muc2 protein found in the intestines of Mpi^{Villin} mice.

16. **The discussion is not referring enough to references since previous results are mentioned by the authors but without citations.**

Response: Citations for the discussion have been added in the manuscript with specific locations described below.

Specific comments

17.**"The presence of this post translational modification primarily in surface and secreted protein, which are involved in many aspects of physiology; hence, modulations in glycosylation may affect many physiologic and disease process" This sentence should be reworded.**

Response: Reworded, and per comment 18, the non-specific disease areas are now left out. Line 75: Glycosylation primarily affects surface and secreted proteins, which are present in several congenital disorders of glycosylation (CDG) such as PMM2-CDG, MPI-CDG, ALG6-CDG, and ALG8-CDG

18. It may be more relevant to focus on glycosylation defect-related diseases observed in the gut rather than immunoglobulin therapeutics or cardiovascular diseases.

Response: Corrected, see comment 17.

19. **There is no verb in this sentence.**

Response: Corrected, see comment 17.

20. It is not clear to what "Mpi phenotype" refers to

Response: This wording has been modified and a more specific description of the intestinal phenotype has been added to the introduction (lines 117-119).

Our analysis revealed that the Mpi-deficient intestinal phenotype may be associated with altered metabolic status, as evidenced by a decreased abundance of fatty acid and metabolic proteins in intestinal tissue lysates

21. **"wrt" should be corrected. This sentence is very long and should be reworded.**

Response: See comment 20.

22. **the "benadryl strain" was not introduced before**

Response: The "benadryl strain" has been taken out of the introduction and described in the methods section (lines 141-145) with citations to the original paper that describes the mutation in detail. Also note, this has been renamed to *benadrl* for trademark infringement concerns.

A candidate *Mpi* mutation (p.H54R) was named *benadrl* and knocked-in as described elsewhere.[10] The *benadrl* allele was isolated by crossing C1 (1st generation CRISPR mice) to C57BL6/N mice then intercrossing the C2 generation to test C3 mice with the homozygous *H54R* mutation.

23. **"phosphatase inhibitor cocktail (Company)" the company is not provided**
Response: Company name "Sigma Aldrich and catalog number" has been updated in the text in Material and Methods section.

24. **All antibodies do not target MUC2**
Response: Correct, sentence updated:

Antibodies were used from Cell Signaling Technology (Table No.1) to detect FASN, ACLY, ACC1 and AeCs , Thermo Fisher Scientific (Table 1), and Abcam antibodies (Table No. 1) were used to detect MUC2

25**. what was the exact concentration of DSS used? This can influence greatly the phenotype**.

Response: We used 2% DSS. This has been updated in line 177.

26. **Details on the vectors should be provided. The culture of HT29-MTX cells is not described.**

Response: The details for the vector and gRNA used to generate LS174T-Mpi KO cell lines have been included. The culture of HT-29 MTx cells have been included in the methods section.

Line 184-187: The culture media for all the cells (HT-29 MTx and LS174T) was supplemented with 10% FBS, 2mM glutamine, 100 units/L streptomycin, 100 units/L penicillin, and LS174T-Mpi KO cell lines 10mg/ml puromycin was added

27. **Typo example: "Tissues from Mpi F/F and MpiΔVillin mice were collected run on (Nupage 4-12% Tris gel) (Invitrogen) for 15 mins"**

Response: Typo has been corrected in Material and Methods section. Line 195.
28. dilution of each antibody should be provided.

Response: Dilution of each antibody added in Table 1.
29. The 16S probe sequence is not provided. I guess the staining was performed on tissues fixed in Carnoy, which is not described.

Response: 16S probe sequence is now provided in Table No.1 and detailed staining for the tissues is now mentioned in the methods section. Lines 244-255.
30. **The Figure 4C mention is not expected here.**

Response: Figure 4C has been removed from the text in manuscript, and placed in the sequential location.

31. **A more specific wording than "visible" should be used. The allergy phenotype is not presented.**

Response: The word "visible" has been changed to "small body size" in the manuscript. The allergic antibody phenotype will be described separately in a different paper, so will not be described further to preserve novelty for a more in depth publication planned.

32. **The reference where this strain was presented before should be mentioned.**

Response: The reference for the JCI Insight publication has been added in the manuscript. (line 291).

33. **the reference of the findings of your group should be provided.**
"The strength of this phenotype most closely resembles Muc2 KO" a word is missing and a reference for the MUC2 KO should be provided.

Response: See above regarding an updated reference (Line 301). The wording has been updated to: The strength of this phenotype most closely resembles the Muc2 KO strain

34. **"fewer goblet cells lead to less mucus and higher levels of neutrophils in Mpi ben/ben" This causality link was not demonstrated at this stage.**

Response: The statement has been adjusted as seen below:

Also, *Mpi* deficiency led to higher levels of peripheral blood neutrophils in $Mpi^{ben/ben}$ at baseline(Fig. 1 J-K).

35. **Why do we jump from figure 1 to figure 3? panels should be reorganized.**

Response: The panels have been updated and cell line data from figure 7 was moved up to this section to demonstrate the relationship between Muc2 expression and Mpi as well. Lines 324-327

36. Where are presented this cell type specific data?

Response: Expression of ER stress markers by immunohistochemistry is now called out in the text for figures 3A and 3C in lines 341.

37. **"The mucus layer is reduced 100 % from 8 µm to 0 µm (Fig. 3 E-F)" figure panels should be reorganized to be presented sequentially.**

Response: We agree, and have arranged this data into Figure 2F-H.

38. **The increase of ER stress in ben/ben mpi mice was presented before.**

Response: The text has been cleaned up so that ER stress is not shown in Figure 1, but Figure 3 all in one place alongside the Mpi^{Villin} data.

39. in which mice strain did you observe apoptosis?

Response: In $Mpi^{\Delta Villin}$ mice small intestine we observed apoptosis. Text updated in lines 348-348-352. The culmination of these cellular stressors is Goblet cell death in the small intestine. TUNEL staining was enriched in Muc2 positive small intestine Goblet cells. 55% of goblet cells were TUNEL positive in Mpi□Villin mice vs. 0% TUNEL positive in co ntrols (Fig. 3E). TUNEL staining was also increased in Muc2 negative enterocytes but to a much lower degree (5% Mpi□Villin vs. 0% WT). In contrast, TUNEL staining was not increased in the colon of benadrl or Mpi□Villin mice (Supplementary Fig 1).

40. Where are these WGA stainings presented? Figure 4A should be indicated.

Response: Fig. 4A has been indicated on line 363.

41. Figure 3G panel should be moved to follow the description in results

We agree this order would be much better and have thus rearranged Figures 2 and 3 substantially along with text describing them.

42. **The magnification provided do not allow to visualize the loss of mitochondria. Where are presented the data related to the size of microvilli? Same question for the small intestine observations.**

Response: The EM images sizes have been increased substantially and white arrowheads indicate where mitochondria are located. Mitochondria were identified by characteristic cristae, which can be seen within the goblet cells and in adjacent enterocytes. Expanded images of the mitochondria and microvilli have been created for the images in Fig 4C.

Similar observations were made for the small intestine microvilli, which have now been included as supplementary figure 2. We hope this provides clear images of the interesting findings.

43. Was apoptosis also increased in the colon goblet cells?

Response: No, apoptosis was not increased in the colon goblet cells. This data was included in the supplementary Fig. 1, and described in the text.

Line 352-353: In contrast, TUNEL staining was not increased in the colon of *benadrl* or Mpi$^{\Delta Villin}$ mice (Supplementary Fig 1).

44. **where are the ben/ben mice apoptosis data presented?**

Response: We did not perform TUNEL staining on ben/ben mice small intestines nor describe it in the manuscript. Given some observations of milder dysbiosis (Supp Fig 2), milder alcian blue staining loss (Lin et al JCI Inight 2025), and no stool blood (JCI Insight 2025), we would not suspect TUNEL staining to be as prominent in this strain.

45. **the verb is lacking**

Response: The whole paragraph has been substantially revised from Lines 359-367.

46. **The IL10 decrease is not significant**

Response: The statement has been corrected and included in the manuscript. Line 402

47. The reference to the figure is not correct, there is a confusion with the other experiments in ben/ben mice...

The Peripheral blood count reference (Fig 5A) has been corrected (line 400) and references to ben/ben mice have been removed.

48. **The microvilli observations are still not presented here for the small intestine. Same comment for goblet cells. All these data should be presented**.

Response: Microvilli EM images for the small intestine have been added to Supplemental figure 2 and zoomed in images have been added for large (Fig 4C)/ small intestine (Supp Fig 3) as described above.

49. **Table 2 is not provided**

Response: Table 2 has been added to the manuscript.

50. **The changes in gene expression are not significant according to what is presented in the figure**

Response: The incorrect statistical test was used, and now using the Welch's test for unpaired, non-parametric comparison of two groups, the corrected p-values are present and in the figure legend.

51. **It is not clear whether proteomics was performed only in the small intestine or also in the colon.**

Response: The proteomics was performed only in the small intestine (n=3 mice/ genotype). Colon proteomics were attempted but the pilot study did not find clear differences like in the small intestine.

52. **where are these data presented?**

Response: This data has been presented in Fig. 7B and Table 2 also included in the text in manuscript (line 437).

53. **The changes in gene expression are not significant according to what is presented in the figure**

Response: See response for item 50.

54. **Is proteomics results (7B) performed in the colon as written in line 340 or in the ileum as written in the figure legend?**

Response: Fig 7B proteomic analysis was performed in the ileum tissue samples as mentioned in the figure legend (7B) and it has been corrected in the text. At the suggestion of another reviewer, proteomics samples were aggregated together and now the results in Table 2 and the text were updated with some new genes and some genes removed (e.g. Aldh9a1, slc25a1, Nnt). However, we kept in some of the genes that were original hits (e.g. Acly), because they came from a higher quality sample (higher proteomic coverage) and were validated on WB (Fig 7F).

55. **Experiments with tunicamycin in HT29 MTX cells are not presented in the methods section.**

Response: The detailed experimental procedure for tunicamycin treated HT29-MTx has been added to the methods section (Line 265).

56. **What type of glycosylation is inhibited by tunicamycin? This information should be added.**

Response: "tunicamycin (inhibits the first step of N-linked glycosylation when UDP-GlcNAc is added to dolichyl phosphate". This statement has been added in lines 472-473.

57. There are no data provided showing that the loss of Mpi was worse than loss of O glycosylation and the comparison with the phenotype of MUC2 KO mice was not evaluated experimentally either.

These comparisons were based on published images of Alcian blue staining of goblet cells, so references have now been provided. If the reviewer thinks this should be included side-by-side in the paper, then we can contact collaborators with access to images from Muc2KO mice and an O-glycan mutant strain (Tvp23b). The statements have now been cited and tempered for balanced interpretation.

Response:
58. **The death of mice 2-3 weeks after deletion of Mpi was not presented in the results.**
Response: We apologize, the UBC-Cre conditional deletion of Mpi is presented in another paper. We have therefore corrected the statement, and this was beyond the scope of this paper.

59. **References should be provided for this mice collection**

Response: Added on line 510: (*Tvp23b, Yipf6, Muc2^{Winnie}*,Myo1d)[21,26,35–37]

60. **Reference for this work should be provided**

Response: Reference 40 was added on line 524.

61. References should be provided

Response: References 21,26, 29, 37-39 been added in lines 511-519.

62. **where was this effect of mannose supplementation reported? There is no reference.**

Response: Reference 18 has been added as Lin et al JCI Insight 2025 on line 545.

63. **Here again no references are provided.**
Response: Reference 48 has been added on line 546.

64. **Supplementary file was not provided**
Response: Supplementary file is now uploaded in the manuscript submission portal, the figures and legends have been updated.

65. **Figure 2G: goblet cells were counted in the colon crypts, not villi.**
Response: In Figure 2G "Goblet Cells/Villus" changed to "Goblet Cells/crypt."

66. **Figure 4E: statistical analyses are not shown**

Response: The statistical analysis have been included in Fig. 4E and all other quantitative panels.

67. **Figure 7D: groups are interchanged**
Response: No groups are interchanged in Figure 7D, Sox9 actually did have higher expression in the mutant mice. Perhaps as a compensatory change or developmental block at this Sox9 expressing stage.

68. **Table 1: all working dilutions of antibodies should be provided for reproducibility.**
Response: All the working dilutions of antibodies have now been provided in Table 1.

69. **Table 3: LS1745 and HT29-MTX were not produced for this paper.**
Response: Thank you for finding this error, sources of LS174T and HT-29MTX are now included in Table 3.

Reviewer #3 (Remarks to the Author)

All the corrections in the manuscript has been highlighted in blue.

Major comments concerning the proteomics part only:

• In your proteomics methods part, you state that you prepared the proteomics samples as previously described by Birchenough et al., yet the publication you refer to did not perform proteomics from gel bands, nor from intestinal tissue, but from mucus, which intrinsically calls for a different sample preparation. You state that gel bands were sent in for proteomics measurement, yet it is unclear why the samples were run on a gel at all, and it is also not clear which bands were excised.

Response: We have updated the methods of the proteomics section to more accurately reflect the methods performed. Our core recommended running tissue lysates for a short distance (1cm) into a gel then excising the entire Coomassie positive top section. References and methods have been updated in the text

• You further fail to provide important information concerning the proteomics experiment, like how many biological replicates were used, how the samples were lysed, if and how they were reduced, alkylated, or digested. You provide no information on the instrumentation (the LC system used, the solvents, chromatography column, gradient, the ion source, the mass spectrometer, or the acquisition mode). You also provide no information about the data analysis of the resulting proteomics data, like which software was used, which database you searched against, your FDR control, your mode of quantitation, your used statistics, etc.

Response: We have consulted with our proteomics core and added all requested details to the methods section now (Lines 193-223), including how 3 biological replicates were used.

• Moreover, the proteomics data should be made available for the reviewers through submission to a proteomics repository (e.g., the PRoteomics IDEntifications (PRIDE) Database by the ProteomeXchange Consortium): https://www.proteomexchange.org/ (like they did in the publication you refer to in your proteomics method section) – this is good practice in the proteomics community.

Response: The proteomics data have been uploaded to MassIVE, this can be accessed by the reviewers using the following accession number: MSV000098815.

• In the results section discussing the proteomics data, you refer to Table 2 (line 312), which is not provided. The plot shown in Figure 7B is not a common way to visualize proteomics/peptidomics data. The names refer to proteins, did you analyze proteins or peptides? Moreover, it is not explained anywhere what the green triangles or red dots in Figure 7B represent. How many proteins/peptides were identified in total? How many of them are differentially regulated, to which extent and significance level (multitesting corrected)? How many biological replicates were used?

Response: We appreciate the reviewer's detailed review and have now uploaded raw data and proteomics data to answer their questions regarding number of peptides (represented as spectral counts, PSM). Table 2 is now included. We have used this visualization method as it allows differential expression to be seen easier across a range of expression as shown in previous publcations (PMID:37339972). The colored dots have been labelled in the figure now in a standard format.

• In lines 336 – 340, you state that you performed proteomics analysis on colon lysates, and refer to Figure 7B, which, according to the legend, is peptidomics of the small soluble fraction of the terminal ileum. Please elaborate which statement is correct.

Response: Proteomics analysis was performed of the whole ileal tissue lysate as stated in Figure 7B legend. This statement has been corrected and incorporated in the manuscript. We apologize for the inconsistency.

Taken together, there is insufficient information about the proteomics experiment to review it properly on a technical level. Furthermore, it is unclear what exactly was analyzed and is shown in Figure 7B, and no additional information (i.e. protein tables) is provided. The proteomics part of this publication does not meet the standard required for publication.

Response: Please see above specific comments addressing each of these issues.

General comment: Overall the manuscript is hard to read, especially the introduction contains only fragments of sentences and lacks references. Mouse

models and abbreviations are not explained upon first mentioning them. The method section (in part, e.g., proteomics/peptidomics, mouse models, primers, antibodies (ATF4 for IHC), table 2 and supplements are lacking.

Response: The manuscript have been revised thoroughly with appropriate explanations of the mouse models and elaborated materials and methods section.

Response to Reviewers Comments #2
We thank the reviewers for their continued work to help improve the quality of this manuscript. Most of the points relate to presentation, clarity of writing, figure legends, and completeness of methods. These technical aspects are fully addressable and will bring up the quality, interpretability and visibility of the work up to the level of Nature Communications.

Through our own continual internal scientific review process, it became apparent that the way qPCR data calculation method did not align with best practices, prompting a review and correction. The previous calculations showed >10 fold changes in magnitude being reported. Now we find much closer alignment of RNA expression levels with some comparisons losing statistical significance. We are committed to transparency and believe this correction reflects our dedication to scientific rigor during peer review. We strive for scientific integrity and honesty and want to correct this error before going to publication. The following data are affected: 3B, 4D, 5E, 7C, D, G. Figure 4B is not affected as it was performed by a different investigator using industry standard methods.

Reviewer #1 (Remarks to the Author):

The authors have addressed our previous comments thoughtfully. Most of our original concerns related to the clarity of writing and interpretation rather than the experimental results themselves, and the revised manuscript reflects meaningful improvements in these areas. The edits indicate that the authors understood our feedback and implemented it effectively.
Reviewer #4 (Remarks to the Author):

I co-reviewed this manuscript with one of the reviewers who provided the listed reports. This is part of the Nature Communications initiative to facilitate training in peer review and to provide appropriate recognition for Early Career Researchers who co-review manuscripts.
We appreciate the comments and efforts of reviewers 1 and 4 to improve the quality of the manuscript.

Reviewer #2 (Remarks to the Author)

Although the authors have addressed most of my previous comments, the quality of the manuscript remains below the standards expected for publication in Nature Communications, particularly with regard to the writing of the introduction, the details of the materials and methods to ensure reproducibility, and the overall care taken in the writing. This manuscript has not been carefully proofread by the authors.

The overall study design is clearer now that another article from the authors was published in JCI Insights (https://doi.org/10.1172/jci.insight.180752) to present the mouse MPI KO models. However, I feel that the submission of the previous version of the current study was pre-matured, since it represented an unnecessary hard reviewing process due to undisclosed information.

The small intestine and colonic phenotype of the MPI hypomorphic and intestinal conditional KO mice was already described in part in the JCI insight article (presence of abnormal goblet cells and lower mucus production). The current study expands these observations, including in an inflammatory context by using DSS model and by searching underlying cellular/molecular changes.

Specific comments:

1. The viability data of the conditional KO should be presented in the methods or results but not in the introduction.
Corrected.

2. Figure 1D: villus should be replaced by crypts (also in figure 2F). Line 806: Replace "CRIPSR Cas 9 targeted mice" by a more specific designation or remove. "Het = heterozygote, Homo = homozygote » are not used in the figure, to be removed.
Corrected in Figure images and legend. "CRISPR Cas 9 targeted mice" removed as it is redundant with the Mpi^{ben} strain described in the methods.

3. Figure 3: the legend is missing in 3D (MPI F/F or villin). Line 851: remove "repeated five times"?
Corrected.

4. Figure 4F and G: statistical analyses are missing for mitochondrial activity data.
Two way ANOVA with tukey post-hoc tests were performed and now statistical measures are added to the figure and legend. Since there are some dose dependent effects of tunicamycin present at certain timepoints. Text has been added to reflect this.

5. Figure 5: all abbreviations should be defined in the legend. Figure 5B: the tissue where these genes were measured should be indicated.
Abbreviations added, and tissue clarified as colon.

6. Figure 6: Line 900: can you confirm this is colon and not small intestine as indicated? 904: should villus tip be corrected to crypt?
Yes, the reviewer is correct in both instances and nomenclature has been updated.

7. Data availability: the database where the data were deposited should be indicated (SRA for microbiome?). Idem for proteomics data.
Data deposition details have been updated with microbiome data going to NCBI and proteomics data deposited at MassIVE.

8. Title: I think that the title should use more specific wording than "host microbe equilibrium" and "gut immune balance". Metabolism is also vague, it should be specified epithelial cell metabolism.
We appreciate the attention given to having an accurate and specific title. This will help future impact of the report. We have made updates to indicate mechanism with

“Microbial containment” and more specific immunity words like “inflammation” instead of immune balance. Lastly, we have added epithelial mitochondrial metabolism to be specific to the epithelial cell type and indicate the type of metabolism affected is mitochondrial.

9. The introduction still lacks clarity. For instance, Lines 108 - 119, it is not clear whether the authors refer to their previous or current publication. Line 47: this sentence suggests that there was a global MPI KO model while it was hypomorphic only at the whole-body level. Line 83: congenital disorders of glycosylation are defined twice. Line 87: there is no verb. Line 94 : the role of MPI was already defined line 87. The introduction should be reorganized since it is still hard to read.
With the help of a copy editor, we have substantially re-written the introduction with a focus on: Starting broad (glycosylation importance), then narrowing to mucins, CDG, MPI, and our mouse model (distinguishing the previous report from this one). We use shorter sentences, clear transitions and end with a strong rationale and study objective.

10. In the material and methods, all suppliers and catalogue number should be provided to ensure reproducibility. Most of them are still missing in the current version.
We have added all catalog numbers in the materials and methods section. These are also included in supplemental tables 1 and 4.

11. Line 182: Did the DSS treatment lasted 5 or 7 days?
We used DSS for 7 days - corrected.

12. The methods for the culture of cell lines are not sufficiently described (lines 189-193). There is no detailed information related to the generation of KO cell lines. All references to commercial products should be provided. “and LS174T-Mpi KO cell lines 10mg/ml puromycin 191 was added” should be corrected.
We have now added significant methods for the generation of the Mpi KO cell lines including viral vector production, infection, and selection.

13. Add MPI ben mice in the microbiome method section (line 195)
Mpi^{ben} Added.

14. L245: the sentence should be corrected: “fluorescently labeled 16s RNA probes conjugated with in Hybridization buffer”. That type of typo are still frequent in the manuscript.
Corrected.

15. Line 263: indicate which cells were used in the searhorse assay.
HT29-Mtx cells were used. (line 275)

16. Line 298: the reference for the scRNAseq data is not appropriate (Uhlen, M. et al. A genome-wide transcriptomic analysis of protein-coding genes in 726 human blood cells. Science (1979) 366, (2019).) The methods used to analyze these scRNAseq data are missing.

We have updated the results to include a different “primary publication” of the human protein atlas: Karlsson M et al. A single cell type transcriptomics map of human tissues. *Sci Adv.* 2021. PMID 28818976

17. Line 315: a space is missing after baseline
Added.

18. Line 301: replace Intestinal by colon to match with the figure 1C and D.
Replaced.

19. As indicated in my previous review, this sentence is misleading “To test whether the colitis phenotype was intrinsic to the colon” (Line 332) because MPI is deleted in all intestinal epithelial cells, not only in the colon.
We have now changed the sentence to: “To test whether the colitis phenotype was intrinsic to intestinal epithelial cells, we created the $Mpi^{\Delta Villin}$ mouse strain.”

20. Figure 2K: the meaning of 136-7 and 1049-8 should be explained in the legend.
Added.

21. Line 341 “terminal” should be corrected
We can defer to copy editors, but in this context C-terminal is an adjective modifying the noun N-glycans, so it is formatted correctly. If it was a noun, then C-terminus would be correct.

22. Figure 4E: the legend is missing (color code in the figure).
A legend has now been added for 4E.

23. Line 399-408: these data related to the spontaneous colitis should be presented together with figure 2 to keep a logical flow.
We have moved this figure to now be figure 3 and moved the corresponding text as well.

24. Stars indicating significance were added in the figure 5B for IL10 when compared to the previous version while you added in the text that this decrease was not significant (Line 408). This is highly confusing on how statistical analyses were performed.
We apologize for the confusion. And now, as described in the introductory paragraph of this revision, the way qPCR data was calculated was incorrect and we now report housekeeping gene normalized qPCR results for all figures. This makes TNF and IL-12 cytokine results not significant and the IL-10 levels are marginally significant now ($p=0.04$). Some of these track change modifications are not visible in the revision as we moved the paragraph according to the reviewer’s suggestion. The figure data points have been updated accordingly to be accurate now and the statistical test (Mann Whitney test for non-parametric data) is included in the figure legend.

25. Line 422-426: the results of the microbiota of MPI ben mice is discussed here, the

divergence between the two models (CKO or ben) should be discussed since it would be expected that the intestinal MPI drives most of the effects.
Yes, the interesting significance of the result is that the CKO model has a stronger version of the microbiome changes seen in the *benadrl* mice. To compare the CKO and ben models more clearly, we have combined the data from Supplemental figure 3 with Fig 6A (supplemental figure 4 is now number 3). This now increases the strength of the F/F group and shows intermediate phenotypes for several bacteria including Proteobacteria and Chlamydiae. (Lines 428-431).

26. Line 429: there is no verb in the title
The title is updated to "Mpi is essential for Paneth cell and granulation." Maturation was taken out as the qPCR results were no long significant for a loss in maturation transcription factors.

27. There is no capital letter in "goblet cell" (to be changed in all the manuscript)
Changed throughout.

28. Line 440: "detect any change in the abundance of the small soluble molecules", I guess you mean protein or peptides?
Yes, clarified.

29. Line 443: why do you only comment the decrease in Reg3g while other AMP are also reduced in qPCR analysis?
This is a good point, and now that we have updated the qPCR analyses and Reg3g is no longer significant. Several other defensins remained statistically significant (or nearly so) but not to the degree of before. Therefore, we have removed references to 100 fold decrease and include descriptions of the other genes tested in the figure.

30. Revise: "compared to wild type. Including –SDH and Ndufa10 all decreased by 10-20- fold (Fig. 7G)." Line 461
As the sample size of the proteomics samples were small (R3 comment) we are removing language that could be perceived as implying statistical significance. The proteomics data was used as a screening method with qPCR and western blot used as confirmation in a more rigorous form. The protein names have also been uncapitalized as this is consistent with mouse protein nomenclature.

31. Line 467-473: In this paragraph it is highly confusing whether these data were obtained in the colon or ileum. You are referring to the same figure for the colon as the one you referred before for the ileum (Figure 7B).
We see how this was confusing and have therefore edited the whole section to clarify that only ileal samples were tested. The figure legend has also been updated. This section also included information about the mitochondrial respiration assays; the text has been moved up to the text surrounding that figure in 5F (formerly 4F) to keep the flow of results in line with the figures.

32. 478: the figure panel 4F should be moved to in figure 7 to follow the description of the results.

We have moved the text up to the section with Figure 5F (formerly 4F) to keep the flow of results in line with the figures. We have also added a reference and call back to that section for the results of mitochondrial protein decreases in Figure 7.

33. 528 “bacteria break down mucin O-glycans or block quarum sensing” I guess you mean quorum sensing but I do not understand the link with the discussion in this context.
Thank you for allowing us the opportunity to improve this section. We have now added text and references that spell out how secreted mucins and their glycans are important for 1) suppressing pathogenic bacteria and 2) promoting bacterial diversity.

Lines 545-549: O-glycans on secreted mucins such as MUC5B can be potent blockers of bacterial quorum sensing to prevent infection by pathogenic bacteria.[40,41] Muc2 glycans potently promote bacterial diversity, perhaps due to shorter, less extended glycans compared to MUC5B and Muc5ac.[42]

34. 565 : Correct : “oxidative phosphorylation 27 ,28 , 29). Glycosylation could impact mitochondrial proteins due to changes in protein folding and stability 30)”
Corrected

35. 571: “Paneth cells secrete anti-microbial peptides stored in dense granules at the base of small 571 intestine villi” In this case, crypt should not be corrected to villi.
Corrected
36. Line 581: Revise: Gfi1 (Stappenbech)
Corrected- reference added.
37. Line 923: Do you mean markers of Paneth cells instead of goblet cells?
Yes, corrected.
38. Line 959: (Top)- there is no bottom panel?
Reference to Top removed.

Reviewer #3 (Remarks to the Author)

Remaining points for the proteomics part only:
We thank the reviewer for helping us make our results clear for readers. We provide this broad answer first then respond point by point below.
We have gone through and reviewed our proteomics data with an expert, Dr. Jim Moresco (former director of proteomics core at the Salk institute). He helped us see that initial two samples tested in 2022 (1 $Mpi^{f/f}$ and 1 $Mpi^{\Delta Villin}$) had several fold more protein measured compared to four samples prepared in 2024. Two different people prepared each batch with the first using mechanical lysis by bead beating, and the second using water bath sonication. The qPCR and WB results used to validate results (Fig 7 C, D, F, and G) all used the sonication method for tissue lysis.

Additionally, after reviewing proteomics methodology section, we recognized the absence of the lipidomics methodology. This has now been addressed by adding a methods section. Furthermore, the lipidomics results (while not statistically significant) were not described in the body of the paper. This has been added in the final section. It has been integrated into the results of Figure 7, which we admit were described in a confusing way. This version conveys greater clarity on which tissue types were used (small intestine ileal sections) throughout, and it indicates how the second set of proteomics data in the supplement complements data in Figure 7B.

1) The information that you just performed a clean-up per gel is still missing in the manuscript. Now it reads as if you would have run a whole SDS-PAGE separation.
We see the confusion and have clarified that gel clean-up was performed by gel electrophoresis.

2) Your number of replicates is very low with 3 mice in a group. In addition, your method description still lacks the information how you did your quantitative analysis and statistics to determine significant protein changes.
Our intention was to perform a screen of potentially altered proteins to find the few proteins that were significantly altered (<10% in mutant) and fit a similar pathway. Therefore, we did not perform statistics but relied upon orthogonal validation for reportable results. Given the two sample different preparation methods for the proteomics samples, we have separated the data into Figure 7B and a supplemental figure. Statistical analyses therefore cannot be performed. Instead, we show results are replicated (low Fasn, Defa22, Defa2, Acly, Acss2) with two different sample preparation methods. Furthermore, even if we were to try and increase the replicate numbers, the $Mpi^{\Delta Villin}$ mice take so long to get by breeding that it would not be feasible to get additional samples within the allocated time.
As consistent with our approach of screening by proteomics, validation was performed by western blot analysis on the selected group of proteins with related function (fatty acid metabolism and defensin peptides).

3) The information of how to access your proteomic data is missing in the manuscript. While you report the accession number, there is no information about where you uploaded the data, and with the accession number alone, there is no way to access the data.
We apologize for this oversight. Information about data repositories has been added. Proteomics data was deposited to the MassIVE database.

4) The visualization in Fig. 7B lacks in providing information about statistical significance of observed changes (in comparison to e.g., a volcano plot). Moreover, the description of the axes appears faulty (brackets wrong in y, why is the intensity in x (which intensity is meant? Or are these spectral counts?) reported as exponential with a base of 2?). The now added Table 2 only lists a few selected targets and it is unclear why these were selected to be reported and whether their abundance change is significant. In the source files one can find the whole protein table which should make it into the

supplement after addition of statistical analysis results which are also missing in the current table.
We appreciate the reviewer’s attention to detail in displaying data. As described above, we did not perform statistics due to the nature of the experiment. We have used plots like with before with Dr. Moresco to depict differences in protein abundance (y-axis) with consideration for the total amount of each protein (x-axis, intensity in arbitrary units). The intensity is integrated for all peptides originating from a given protein. We have now included this analysis methodology to the methods section.

Regarding the table, protein selection is described above. Also, we do not call protein changes as significant from proteomics, so as to not imply statistics have been applied; instead, we rely on the validation methods of western blot or qPCR (performed on the second set of samples).

The whole protein table is provided and is separated for the data presented in Figure 7B and the supplement.

<table>
<tr><th>Reviewer</th><th>Line</th><th>Comment No</th><th>Comment</th><th>Author Response</th></tr>
<tr><td colspan="5">Note to Reviewers: We sincerely appreciate the time reviewers have taken on this extended review process to improve the quality of the manuscript. Already, we can tell that it will have a meaningful impact to the community and literature broadly, as evidenced by 2 citations of the pre-print article (including one in Nature Reviews Gasteroenterology & Hepatology).

We reflected on the constructive feedback and reached out for professional scientific writing/ copy editing assistance to improve the readability of the report to make it suitable for publication in Nature Communications. Wording was changed in several places as was an original criticism of several reviewers. They helped assemble this matrix format to respond to critiques specifically and clearly.</td></tr>
<tr><td>2</td><td></td><td>1</td><td>As explained in their letter, the authors have re-analysed the qPCR data and identified calculation errors in several figure panels (3B, 4D, 5E, 7C, D and G). In several cases, statistical significance has been lost compared to the previous version. In addition to the changes in qPCR calculations mentioned by the authors, I noticed that the number of samples/dots per group (n = 5) in the new Figure 3 (formerly Figure 5) is different to that in the previous version (n = 7). Furthermore, the legend of Figure 3 still states that n = 7 (line 1029). The authors should explain why data were excluded between the two versions.</td><td>We highly value the critical points reviewer has raised in the comment and we acknowledge that it’s our mistake and have a clear explanation for this. Since the data was aggregated across multiple experiments, during recalculation of the mRNA expression one run was not included mistakenly. In the current revised version, we have included the missed data points and have n=7 for IL-12 and TNFa, but only n=5 data points for IL-10 with matching GAPDH could be recovered in Figure 3B. We have also removed bar plots throughout to align with Nature figure standards.
Figure 4D: To make the results comparable to 4B, we calculated 4D as Fold change by the ΔΔCt method with GAPDH as the control housekeeping gene. Independent biologic replicates for $Mpi^{f/f}$ and $Mpi^{\Delta Villin}$ data points were added and figure legend was updated accordingly. Now with increased power, we can see the results of Mpi^{ben}+ DSS shows similar increases in ER stress as $Mpi^{\Delta Villin}$ at baseline.
Figure 5e. No changes.</td></tr>
</table>

2	902, 916<br><br>905<br><br>537-542	2	Similarly, in Figure 7C, there are only 5 dots in the graphs but n=7 is indicated in the figure legend (line 1122). The number of replicates also changed compared to the previous version in Figure 7G, for instance for FASN there was 7 replicates for the control and 5 replicates for the KO, while in the new version there are 6 replicates in each group. Does this mean that samples were mixed between groups in previous version?	We thank the reviewer for highlighting this. Reanalysis of the original qRT–PCR Cq files revealed that the discrepancies in Figure 7C now Fig 7g resulted from figure assembly rather than experimental or pre-analytic sample-handling issues.<br>The raw data review confirms six independent biological replicates per genotype ($Mpi^{F/F}$ and $Mpi^{\Delta Villin}$), with no sample mixing, reassignment, or outcome-based exclusion. All figures and legends have been corrected to accurately reflect the underlying datasets.<br><br>Similarly, some data points in **7d** (formerly 7C) did not have corresponding GAPDH performed, so could not be included in the graph. Figure legend has been updated to show the 3-6 replicates present (line 905). As qPCR data of Paneth cell maturation was no longer statistically significant, we have removed corresponding discussion points in lines 537-542. Reference 52 has been removed here but due to difficulties in track-changes in reference manager software, we will change reference numbers in post-production if article is accepted.<br><br>**Figure 7** panels were rearranged to follow the order of the text. Figure legend updated according to the number of replicates shown.
2		3	In the new version, the y axis of some qPCR data is 'Fold change compared to Gapdh', but this wording should be	We completely agree with the reviewer and as suggested we have corrected "Fold change compared to Gapdh" to "mRNA

			corrected to 'mRNA level' or 'mRNA expression' since the expression level is normalized to Gapdh but there is no fold change calculation.	level" in the y-axis of the qPCR data.
2	280	4	"This strain was previously described" a reference should be provided.	In line 280 reference has been included for "This strain was previously described" comment.
2	285<br><br>288-297	5	correct "havespontaneous colitis"	We have corrected the sentence to "The Mpi$^{\Delta Villin}$ mice developed spontaneous colitis with a much smaller body size and shortened colon (Fig. 2 a-d)."<br><br>The rest of the paragraph was revised to keep figure panel descriptions aligned with the text and improved for readability by the copy-editor.
2	333	6	The culmination of these cellular stressors is goblet cell death in the ileum' and line 378 'the cumulative effect of Mpi deficiency is higher ER stress and some goblet cell apoptosis in the ileum', the structure of this repetitive paragraph should be improved.	We thank the reviewer for this helpful suggestion. We have revised the paragraph to remove redundancy and improve structure. We have removed both sentences and included, "These findings indicate that Mpi deficiency results in elevated ER stress and selective goblet cell apoptosis in the ileum."
2		7	Figure 5F and G: The representation of statistical results does not show which groups are significantly different.	This has been corrected and explained in the figure legend of Figure 5f and g using "+" and "#" as placeholders.
2		8	Figure 6A: the lower panel is hidden by the Figure 6B.	The Figure has been rearranged to make the lower panel of Figure 6a visible.
2	392, 395	9	'The Mpi ΔVillin phenotype had larger microbial shifts than the Mpi ben microbiome but the compositional changes were similar', this conclusion is not valid since there is no significant differences	We have removed both statements.

			between the control and Mpi ben mice. Line 430: "indicates they are more sensitive to mucus regulation» this statement is over speculative and should be removed.	
2	415-416	10	"terminal ilium » to be corrected (idem line 450).	In Line 415 "terminal ilium" has been corrected to "ileum". Also in line 416 corrected to "ileum" in current manuscript.
2	760, 784	11	In figure 1D and 2F: villus should be replaced by crypts since there is no villi in the colon. I have already requested this change in the previous version but the authors did not apply it.	We thank the reviewer for reiterating this point. The terminology has now been corrected in Figures 1d and 2g, with "villus/villi" replaced by "crypt/crypts", which accurately reflects colonic architecture.
2	262	12	the reference for the single cell atlas has not been changed. The is a PMID (28818976) written in the text and it is not a relevant publication.	We have deleted the PMID 22818976 written in the text, and added the citation for tissue scRNA-Seq dataset from the Protein Atlas publication.
3	195-197		The experimental design of the proteomics part is rather poor with n=1 and two years later n=2, with the main proteomics figure (7B) being based on a single experiment. Your two statements in your rebuttal that you did not perform statistical testing on the proteomics data and that you performed a screen of potentially altered proteins that were significantly altered (<10% mutant) and fit a similar pathway are contradictory. If you did not perform statistical testing you cannot determine significantly altered proteins. The method for quantitation is not clear. Did you use ion intensity (Fig. 7B) or spectral	The proteomics was exploratory and not powered for formal inference. Repetition of the results showed consistently altered proteins in related biological pathways, which caught our eye. The candidate proteins were then subjected to validation studies by WB and qPCR as shown. Importantly, all major biological conclusions were drawn from independent orthogonal validation using qPCR and Western blotting, which confirmed several key proteomic observations. We now explicitly state that the proteomics data served to guide these targeted validation experiments rather than to stand alone as evidence of differential protein abundance.

	188-190<br><br>192-193		counting (Suppl. Fig. 3A) for quantification?	We did not perform statistical hypothesis testing on the proteomics datasets, and we agree with the reviewer that, as originally written, our use of the term “significantly altered” was imprecise. We apologize for the imprecise use of language.<br><br>Our quantitation method has been clarified: Intensity is defined by MaxQuant as “Summed up eXtracted Ion Current (XIC) of all isotopic clusters for the identified AA sequence (protein)”. Line 188-190<br><br>Re-examination of the proteomics metadata revealed that the samples were processed within the same month rather than years apart; accordingly, lines 192–193 have been corrected. Importantly, despite this correction, the observed differences in protein abundance remain, and the remainder of the interpretation is unchanged.
3	449	1	I would not call this lipidomics or even a lipidomics screen as you did in the main manuscript (line 488), but total fatty acid profiling, since you hydrolyzed all complex lipids and analyzed just the total fatty acids, as described in the supplement, lines 1201-1236.	We have replaced the words “lipidomics screen” with “total fatty acid profiling” in line 449 as suggested by the reviewer.
3	446, 988	2	Please clarify whether the fatty acid profile you report in supplemental figure 3 is generated from ileal tissue (line 488) or serum (line 1206).	The fatty acid profile we reported is generated from the ileal tissue (Line 449) not from serum (Line 988). The statement in Line 988 has been corrected to ileal tissue lysate.

3		3	Figure 3C: What does the nd above the bars mean? Do you mean ns, non-significant? Please correct.	Fig 3C has been corrected and ns is now used consistently to mean not significant. No significant results were found in Suppl. Fig 3 c-e.
3	428-434	4	Please correct Acyl-CoA to Acetyl-CoA and delete triglycerides. You only show fatty acid de novo synthesis not triglyceride synthesis proteins.	We have modified the sentence to read:<br>"Many of the altered proteins are enzymes involved in acetyl-CoA metabolism and fatty acid de novo synthesis, as well as components of mitochondrial metabolism..."<br><br>We have also corrected the sentence to:<br>"Furthermore, enzymes essential for converting Acetyl-CoA to fatty acids (Acly, Acc1, Fasn)..."
